# Evaluation of peptide designing strategy against subunit reassociation in mucin 1: A steered molecular dynamics approach

**DOI:** 10.1371/journal.pone.0183041

**Published:** 2017-08-17

**Authors:** J. Lesitha Jeeva Kumari, R. Jesu Jaya Sudan, C. Sudandiradoss

**Affiliations:** Department of Biotechnology, School of Biosciences and Technology, VIT University, Vellore, India; University of Akron, UNITED STATES

## Abstract

Subunit reassociation in mucin 1, a breast cancer tumor marker, is reported as one of the critical factors for its cytoplasmic activation. Inhibition of its heterodimeric association would therefore result in loss of its function and alter disease progression. The present study aimed at evaluating peptide inhibitor designing strategies that may serve as antagonist against this receptor-ligand alliance. Several peptides and their derivatives were designed based on native residues, subunit interface, hydrogen bonding and secondary structure. Docking studies with the peptides were carried on the receptor subunit and their binding affinities were evaluated using steered molecular dynamics simulation and umbrella sampling. Our results showed that among all the different classes of peptides evaluated, the receptor based peptide showed the highest binding affinity. This result was concurrent with the experimental observation that the receptor-ligand alliance in mucin 1 is highly specific. Our results also show that peptide ligand against this subunit association is only stabilized through native residue inter-protein interaction irrespective of the peptide structure, peptide length and number of hydrogen bonds. Consistency in binding affinity, pull force and free energy barrier was observed with only the receptor derived peptides which resulted in favorable interprotein interactions at the interface. Several observations were made and discussed which will eventually lead to designing efficient peptide inhibitors against mucin 1 heterodimeric subunit reassociation.

## 1. Introduction

Protein–ligand interactions play an important role in many biological processes. Notably, membrane receptors are the starting point for a huge variety of cellular signal transduction pathways [1]. Interactions between ligands and their membrane receptors affect all aspects of cell behavior, be they related to tissue patterning, cell differentiation, cell growth, or cell death. The engagement of a cell surface receptor by its specific ligand(s) initiates a signaling cascade that ultimately culminates in changed patterns of gene expression, thereby altering cellular characteristics [2]. Mucin 1 is one such protein that participates in signaling cascades through interaction of the membrane receptor with its highly specific ligand. MUC1 overexpression was associated with a recurrence and distance metastasis in breast cancer [3]. These breast mucins are expressed by malignant epithelial cells and they elicit an immune reaction. The up-regulation of mucin expression associates with tumor invasion, by reducing cell-cell interaction facilitating cell detachment [4]. The determination of the level of MUC1 protein in the serum has been exploited as a measure of tumor burden and changing levels, as a reflection of the response to therapy [5]. Among the various mucins expression in breast cancer, MUC1 is reported as potential prognostic marker, having the strongest relationship with patient outcome and a potential target for therapeutic interventions in breast cancers [5,6].

Mucin 1 is a dimeric molecule containing two subunits namely alpha and beta formed as a result of an autoproteolytic cleavage at the “G^+1^S^-1^VVV‟ consensus site in the SEA domain [7,8]. Following the cleavage the alpha and beta subunits reassociate forming a receptor-ligand alliance through noncovalent interactions and can subsequently elicit a signaling cascade [2,9–12]. The elucidation of signaling cascade is vital in mucin 1 due to its involvement in cell development, differentiation and adhesion [9]. The sequences, N-terminal to the “G^+1^S^-1^VVV” cleavage site, within the SEA module, are predicted to be essential components of the subunit association since no binding was observed when 12 amino acids were deleted downstream towards the N-terminal of “G^+1^S^-1^VVV” cleavage site. These studies provide a strong support that this peptide sequence could serve as a target site for the rational design and identification of molecules that will act either as agonists or antagonists and inhibit the binding of the cleaved partners thereby alter signal transduction and, hence, cellular behavior [2]. This model could provide the basis for modulating cell phenotype by selectively intervening in the alpha-beta subunit interaction [11]. The generation of ligand-receptor alliances by SEA module-mediated cleavage of membrane-associated mucin proteins is an emerging theme [13]. Exploiting the advantage of *in silico* studies over *in vitro* in predicting drug interactions and validating drug targets in terms of time and labor and elimination of false positive and true negatives, a docking study and simulation studies were carried out. Promising results of this work will help in identifying ligand(s) that can serve as leads in designing potential inhibitors of mucin 1- SEA domain cleavage.

Peptide ligands have tremendous therapeutic potential as efficacious drugs [14]. In addition, peptides possess several attractive features when compared to small molecule and protein therapeutics, such as high structural compatibility with target proteins, the ability to disrupt protein-protein interfaces, and small size [15]. The conventional approach for designing peptide vaccine against a particular disease involves stimulation of the immune system using the whole pathogen responsible for the disease [16]. Nevertheless peptides that modulate protein-protein interactions (PPIs) can be directly derived from the crystallographic interface of PPI, or from the screening of peptide sequences that do not originate from natural proteins using phage display library screening protocol [17]. Researches now are largely focused on the incorporation of stereochemically constrained amino acids into peptides, which effectively reduce the difficulties with peptide chain conformations, enhance receptor selectivity and pharmacokinetic properties [18]. Considerable progress has been made in the development of novel *in silico* tools to design therapeutic peptides [16]. An *in silico* peptidome is usually predicted through the use of machine learning algorithms or are a direct stretch of a protein's subsequence, since peptides are more selective being derived by the linear protein sequences [19, 20]. A combinatorial approach involving in formation of protein's interface with sequence alignment serves as yet another important step in theoretical design of potential peptide inhibitors [21]. In addition computational methods combining molecular dynamics simulations and binding energy calculations could give both the structural and the energetic perspective of peptide inhibitors to evaluate the binding affinity of peptides [17]. Since protein-interface-derived peptides constitute the major source of rational design and since the interacting partners are protein molecules, we focused on designing peptide based inhibitors [15].

In the present study the hetero dimeric structure of mucin was selected as the source structure of which the alpha subunit was treated as receptor and the beta subunit as the ligand. Peptides were designed using several approaches, which included building peptides from receptor interface residues to ensure specific binding. Secondary structure specificity was also tested using peptides designed into specific secondary structure. Binding affinity of peptide residues at every position was used to generate derivative peptides through mutation. Modeling of all the peptides was done *ab initio* and the dockings were evaluated using molecular dynamics simulation, steered molecular dynamics simulation and potential of mean force (PMF) calculations.

## 2. Materials and methods

### 2.1 Dataset

The target protein was retrieved from Protein Data Bank as Solution structure of the SEA domain of human mucin 1 (MUC1) (PDB ID: 2ACM). This represented the solution structure of mucin 1 SEA domain which is characterized by 2 chains designated as A and B. The lengths of the chains are 66 residues for chain A positioned at 1041–1097 and 55 residues for chain B positioned at 1098–1152 in the full-length sequence of mucin 1. While chain A is extracellular, chain B is noted to be membrane bound through the transmembrane domain and is intracellular. Chain A associates with chain B through intermolecular hydrogen bonds, which is represented as receptor-ligand alliance. From the heterodimeric complex the continuous stretch of residues in the ligand subunit (chain B) that interact with the receptor subunit (chain A) was selected as the reference peptide for evaluation of subsequent dockings.

### 2.2 *In silico* peptide designing and docking

Peptides for the inhibition of subunit reassociation in MUC1 were designed employing several approaches which include a) reference based peptides, b) interface based peptides, c) hydrogen bond based and d) property based peptides. Each of the designed peptides was further validated for anticancer property using Anti-CP program [22] and its bioavailability using Peptide ranker [23]. All peptides that showed a value >0.9 in peptide ranker were considered as biologically active peptides. Peptides with preferred properties were modeled in Pepfold [24]. The best 3D model was selected according to PEP-FOLD server, considering the lowest energy model that indicates peptide stability [25]. The modeled peptides were individually subjected to docking using Cluspro [25,26]. The interface residues GLN1083, GLY1084, GLY1085, LEU1087, SER1090, LYS1093, ARG1095 and GLY1097 between the two subunits were selected as binding site for all peptide dockings in Cluspro. The docked solutions were further refined using Firedock program [27] and the dock scores were obtained. For comparative studies, docking for the reference peptide was carried out using the stretch of the 9-mer peptide “1098SVVVQLTLA1106” interacting with the receptor subunit in the native structure. The Cartesian coordinates for the 9-mer peptide was obtained from the structure of MUC1 (PDB ID 2ACM) in order to study the interaction in its native state.

#### 2.2.1 Generation of candidate peptide derivatives

A systematic mutation approach was employed to design peptide derivatives. The best docked peptides were selected and were mutated one residue at a time replacing them with similar amino acid groups. This peptide library was re-docked with the receptor. The mutations that result in a more favorable global energy were selected and the mutations were subsequently used in combinations to design second generation peptide libraries. In a similar manner subsequent peptide libraries were generated until a peptide that exceeds the affinity of the reference peptide was identified.

### 2.3 Molecular dynamics simulation

The extent of affinity between the peptide and receptor was evaluated using a short simulation of the complexes. Molecular dynamics simulations were performed using Gromacs 4.5 program [28]. OPLSAA force-field parameters were used for all simulations. The interaction time-steps were set to 2fs throughout the entire simulation time. The long range electrostatic interactions were calculated using Particle-Mesh Ewald method with an interpolation order of 4 and maximum Fast Fourier Transforms (FFT) grid spacing of 0.16nm. The coulomb radius was set to 1nm and the short range interactions defined as van der waals was set to a cut-off of 1.0nm [29,30]. Periodic boundary conditions were applied and the bonds were constrained using LINCS algorithm to their equilibrium position. The system was introduced into a cubic box and solvated by explicit solvent using the SPC216 water model and neutralized by adding Na^+^ ions. The system was minimized for 50000 steps using the steepest descent method and subjected to equilibrium at constant temperature and pressure of 300K and 1 atm respectively. The production simulation was performed in isothermal-isobaric conditions for 5ns and the coordinates were saved every 2ps.

### 2.4 Steered molecular dynamics simulation

In SMD simulations, a time-dependent external force is applied to the peptide to facilitate its unbinding from the protein, which cannot usually be achieved by standard MD simulation [31]. The final conformation obtained from the conventional MD simulation was used as the input for subsequent SMD simulations [32]. SMD simulations with constant velocity were then performed. In this process, the receptor was kept as the immobile reference (chain A) and the external steering forces were applied to the reference peptide, defined as chain B and candidate peptides chain C to pull the peptide along the predefined direction. During the SMD simulations, the force was only applied along the pulling direction [33]. The spring constant was set to the value of 2000 kJ mol-1 nm-2 and the pulling velocity to 0.0008 nm ps-1 [34]. The time length for each simulation was set to 5ns to ensure the complete unbinding of the peptide from the receptor. The trajectories were saved for every 1 ps, and steering forces were recorded every 10 fs.

### 2.5 Umbrella sampling and determination of PMF

Umbrella sampling was used to obtain the PMF for each of the peptide binding configurations while varying the inter-protein separation [35]. A series of seed configurations along reaction coordinate obtained from steered molecular dynamics simulations were selected as initial configurations. The reaction coordinate is chosen as the distance between the center of mass (COM) of the receptor and the COM of the peptides along the axis in this case the y-axis. The total number of sampling windows accounted to 146 with a spacing of 0.2nm followed by an umbrella sampling simulations of 730ns corresponding to an average of 13 windows for each peptide and 5ns simulation for each window. In each of these simulation windows we applied a harmonic umbrella potential with a force constant of 2000kJ/mol/nm2. The PMF was calculated using WHAM and the dissociation energy was evaluated as the difference in energy between the plateau and energy minimum along the PMF curve [36].

## 3. Results

### 3.1 Preparation of the receptor

The heterodimeric structure of MUC1 was retrieved from PDB. The structure represented an NMR model, which had 15 ensembles of the heterodimeric receptor molecule. The representative ensemble was selected by calculating the RMSD between each conformation from which the conformer which had the lowest RMSD with other ensembles was selected. S1 Table shows the pairwise RMSD between the ensembles and its identical partner, if any. From the values in S1 Table we observed model 8 and 14 to have the minimal RMSD and were identical to each other. Hence model 8 was used as the reference structure for further studies. Of the two domains, chain A, which represents the cytosolic domain of the protein was treated as the receptor. Prior to docking, chain B which is the ligand, was removed from the complex and the 57 residue monomer (chain A) was subjected to a short molecular dynamics simulation for 5ns to obtain a stable conformation of the receptor. The lowest energy conformation of the structure was obtained and used as receptor for all the peptide docking studies.

### 3.2 Peptide modeling and docking studies with reference based peptides (RB)

**Fig 1:**
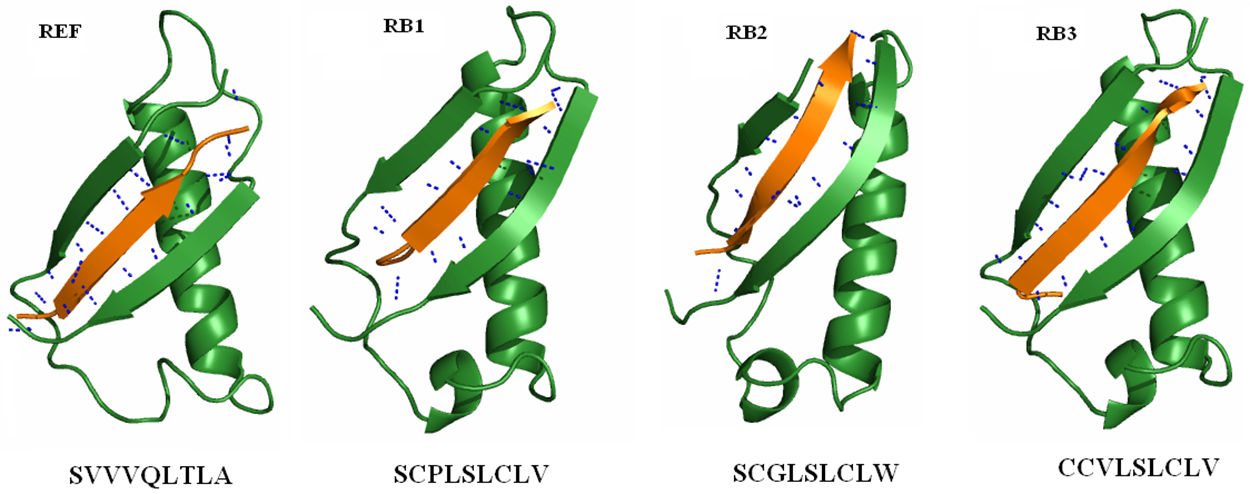
Binding modes of reference based peptides (RB). Colors in the figure represent peptide (orange), receptor (green) and hydrogen bond as blue dashed lines.

The 9mer peptide sequence “SVVVQLTLA”, was used as the reference peptide and was docked at the interface. The reference peptide was then subjected to point mutations obtained from previously published data [37] and the combinations used are reported in S2 Table. The mutants were subsequently subjected to docking with the receptor using Cluspro. Table 1 gives the dock scores for the reference and its mutants. All mutants that exceed the dock score of the reference were identified. From the dock scores of the mutants (Table 1) we identified substitution of residues PRO at 3^rd^, LEU at 4^th^, SER at 5^th^, CYS at 7^th^ and VAL at 9^th^ positions to produce a higher dock score than the reference. Therefore these residues were substituted in reference peptide to obtain a derivative peptide “SVPLSLCLV” (RB1). Derivative peptides from RB1 resulted in RB2 (SCGLSLCLW) and RB3 (CCVLSLCLV). The peptide derivatives were docked and their dock scores and poses are shown in Table 2 and Fig 1 respectively. The third derivative peptide resulted in a global energy of -187.23 closer to the reference peptide. This global energy was poor compared to the reference peptide (Table 1). As we failed to obtain a high global energy score with the reference based peptides, we opted to design peptides from the interface residues.

**Table 1:** Dock scores of the mutant reference peptides.

Peptides	Global Energy	Attractive VdW^a^	Repulsive VdW^a^	ACE^b^	HB^c^
REF^d^	-198.35	-48.82	1.42	-48.82	-4.4
3P	-200.5	-47.98	5.08	-47.38	-5.82
4L	-206.63	-45.96	5.21	-49.11	-8.86
5S	-203.66	-46.12	3.22	-49.45	-8.27
7C	-200.28	-47.68	19.98	-53.29	-7.49
9V	-214.01	-52.83	9.05	-51.39	-4.08

^a^Vanderwaals,

^b^Atomic Contact Energy,

^c^Hydrogen Bond,

^d^Reference peptide.

Peptide names represent the position of mutation followed by the single letter code of the substituted aminoacid

**Table 2:** Docking scores of reference based peptides.

Peptide	Sequence	Global Energy	Attractive VdW^a^	Repulsive VdW^a^	ACE^b^	HB^c^
REF^d^	SVVVQLTLA	-191.84	-49.73	7.37	-47.12	-8.22
Derivatives peptides						
RB1^e^	SVPLSLCLV	-171.07	-39.48	4.18	-45.28	-2.55
RB2^e^	SCGLSLCLW	-136.25	-35.9	2.65	-37.41	-3.93
RB3^e^	CCVLSLCLV	-187.23	-38.64	0.19	-52.15	-6.66

^a^Vanderwaals,

^b^Atomic Contact Energy,

^c^HydrogenBond,

^d^Reference peptide,

^e^Reference Based peptide

### 3.3 *In silico* designing of peptide inhibitors

The initial peptide sequence was selected based on the interacting residues at the interface of the heterodimer. The interacting residues predicted using DIMPLOT module of ligplot [38] are listed in S3 Table. From the table the residues that were preferably opted for the peptide designing include phenylalanine for π-π stacking, histidine, asparigine, glutamic acid for hydrogen bonding and glycine to improve the flexibility of the peptide. The initial peptide sequence constituted about 9 residues [15] which are represented as “GFHNFRQFG”. To validate the positioning of residues within the peptide, the sequence was subjected to peptide scrambler which produced 1000 different combinations with all possible combinations of the residues. This overall procedure resulted in an initial peptide library consisting of 49 anticancer and biologically active peptides. The peptides modeled using PEP-FOLD are displayed in S1 Fig. Intra peptide interactions of few peptides resulted in a helical or sheet conformation, nevertheless most peptides resulted in a coil like structure.

#### 3.3.1 Peptide modeling and docking studies with binding energy based peptides (BE)

**Fig 2:**
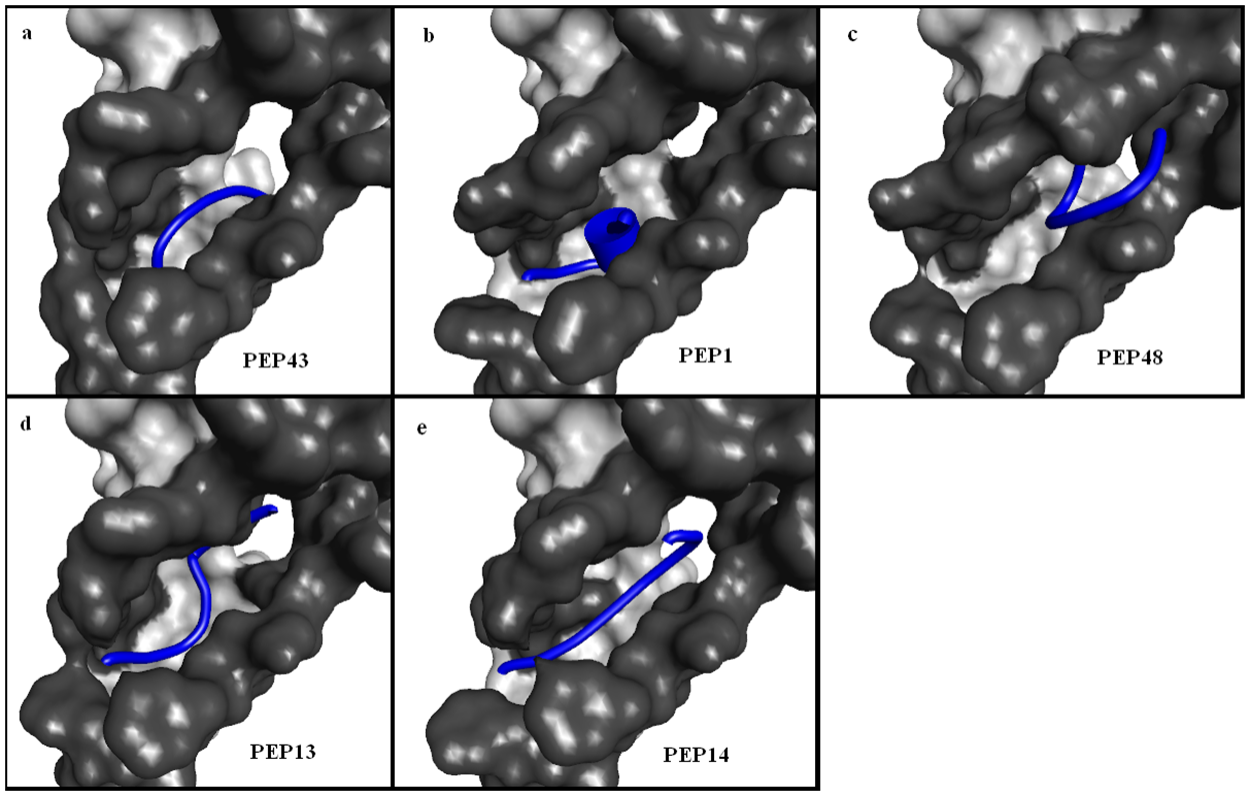
Docking poses for top scoring solutions based on binding energy. The figure shows the interface regions highlighted in dark grey and the peptides in blue.

The best docking scores of the initial peptide library are given in Table 3. From the table it is noted that PEP43 (QGRHFWFFG) has the lowest global energy of -169.01kcal/mol among the 49 peptides subjected to docking. The docked poses of the top 5 best binding peptides is given in Fig 2. The figure shows that, despite good docking scores, the peptides PEP43, PEP1, PEP48 and PEP13 (Fig 2a–2d) failed to completely occupy the subunit interface unlike the reference peptide (Fig 1). However PEP14 (Fig 2e) shows favorable interaction with the subunit by occupying the complete interface due to its linear conformation. Further, the docking results were compared with the dock scores of the reference peptide sequence given in Table 1. As noted from the Table 3, all peptides in the initial library show a poor binding energy than the reference peptide. Taking into account the interface site and minimal difference among the dock scores of the peptides (Table 3) PEP14, represented by “GHQWFRFGF”, was selected as the optimal peptide candidate and was subjected to point mutation using Discovery Studio.

**Table 3:** Top scoring docking solutions of initial peptide library.

Peptides	Sequence	Global Energy	Attractive VdW^a^	Repulsive VdW^a^	ACE^b^	HB^c^
PEP43	QGRHFWFFG	-169.01	-59.81	2.04	-33.66	-6.85
PEP1	FGQHFWGRF	-165.54	-63.84	6.79	-32.13	-4.92
PEP48	WGGHFNFRF	-162.81	-61.98	1.68	-35.5	-3.13
PEP13	GHQWFFGRG	-161.32	-57.21	4.2	-34.85	-4.38
**PEP14**	**GHQWFRFGF**	**-160.11**	**-56.37**	**3.91**	**-40.5**	**-4.1**

^a^Vanderwaals,

^b^Atomic Contact Energy,

^c^Hydrogen Bond,

Subsequent mutations of individual residues in the candidate peptide, resulted in a library of 26 mutant peptides from which we obtained the docked solutions and the results are displayed in Table 4. Unfortunately all the docked scores were lower than the reference peptide. As a result, all mutants that showed a global energy closer to the candidate peptide (PEP14) was selected and subjected to combinatorial mutations to generate the second generation peptides. Table 4 shows that substitutions at positions 2, 4, 5 and 6 reduce the global energy, nevertheless has increased the hydrogen bond score. Therefore all mutants other than 4Y were considered for subsequent analysis. The second generation peptides were generated by substituting position specific mutations in combinations of 2, 3, 4, and so on. This resulted in 84 peptides and upon anticp and bioactivity analysis we arrived at 38 peptides. All the second generation peptides were docked and validated similar to the native peptides and the dock scores are listed in Table 5.

**Table 4:** Dock scores of best mutants from first generation peptides.

Peptides	Global Energy	Attractive VdW^a^	Repulsive VdW^a^	ACE^b^	HB^c^
PEP14	-160.11	-56.37	3.91	-40.5	-4.1
**PEP14 mutants**					
1P	-182.78	-59.86	1.22	-46.2	-3.74
2R	-157.92	-57.41	5.25	-34.17	-4.01
3C	-171.72	-60.26	4.85	-39.38	-5.04
4Y	-143.87	-55.26	6.33	-32.03	-3.13
5W	-156.01	-51.19	2.57	-38.96	-5.99
6H	-158.57	-48.58	0	-36.77	-7.97
7W	-165.17	-53.28	1.66	-40.61	-7.39
8V	-196.61	-62.8	1.76	-43.13	-3.63
9Y	-162.66	-62.53	5.78	-34.94	-3.27

^a^Vanderwaals,

^b^Atomic Contact Energy,

^c^Hydrogen Bond.

Peptide names represent the position of mutation followed by the single letter code of the substituted aminoacid

**Table 5:** Dock scores second generation peptides.

Peptides	Global Energy	Attractive VdW^a^	Repulsive VdW^a^	ACE^b^	HB^c^
PHCWWHWVF	-203.31	-66.33	0.89	-53.42	-2.26
PRCWWHWVF	-192.42	-60.34	5.63	-48.23	-5.58
GHCWWHWVF	-188.88	-60.9	1.62	-51.75	-2.88
PHCWWHWGF	-184.01	-61.11	2.13	-48.11	-3.68
GRCWWHWVF	-183.46	-57.63	3.27	-46.3	-4.34
PHCWWHFGF	-182.64	-59.97	3.64	-47.76	-4.27

^a^Vanderwaals,

^b^Atomic Contact Energy,

^c^Hydrogen Bond.

The peptides PHCWWHWVF and PRCWWHWVF were observed to be the best binding peptides with a global energy of -203.31 and -192.42 kcal/mol respectively (Table 5). In comparison with the reference, the global energy of the peptide PHCWWHWVF-protein complex was higher and therefore was subjected to further mutations to improve the peptide-protein binding affinity. The third generation library consisted of about 63 peptides that resulted in 251 docking solutions. About 5 peptides resulted in a global energy higher than the reference peptide (Table 6). Among the 5 peptides, PHCWWLWVF was observed to be the best with a global energy of -214.75 kcal/mol. Subsequent mutations of the peptide to yield the fourth generation peptides reduced the global energy scores.

**Table 6:** Dock scores of third generation peptides.

Peptides	Global Energy	Attractive VdW^a^	Repulsive VdW^a^	ACE^b^	HB^c^
PHCWWLWVF	-214.75	-62.99	2.63	-55.57	-4.96
WHCWWHWVF	-207.43	-66.88	3.84	-52.06	-4.71
PHCWWYWVF	-204.71	-62.5	3.64	-53.61	-3.56
PRCWWHWVF	-204.28	-62.23	0.19	-46.75	-4.9
PHPWWHWVF	-200.35	-61.44	0.67	-50.19	-1.57
PNCWWHWVF	-199	-62.57	4.34	-48.9	-5.33
PHCWWHWIF	-188.89	-60.13	3.19	-46.88	-4.95
PDCWWHWVF	-188.73	-56.14	3.44	-49.28	-4.77
PLCWWHWVF	-183.05	-54.95	1.4	-45.97	-4.19

^a^Vanderwaals,

^b^Atomic Contact Energy,

^c^Hydrogen Bond

#### 3.3.2 Peptide modeling and docking studies with hydrogen bond based peptides (HB)

From BE peptide dockings we observed that, the hydrogen bond scores of the candidate peptide and derivative peptides were weaker than the reference peptide. Therefore we opted to select the peptides with high hydrogen bond score from the dockings of the initial peptide library, not taking into account the global energy scores. Peptides showing high hydrogen bond scores are given in S2 Fig. We identified PEP41 (QGHRFWFFG) as an optimal peptide as it occupied most of the interface region and also poses a high hydrogen bond score closer to the reference peptide. Derivative peptides were therefore designed by performing point mutation on PEP41 similar to the REF and BE peptides. Subsequent mutation on PEP41 yielded peptides with higher global score, however the hydrogen bond scores for all the derivative peptides were lower than that of PEP41 as shown in S4 Table. Thereforefurther mutational studies were not carried on PEP41.

#### 3.3.3 Peptide modeling and docking studies with property based peptides (PRP)

**Fig 3:**
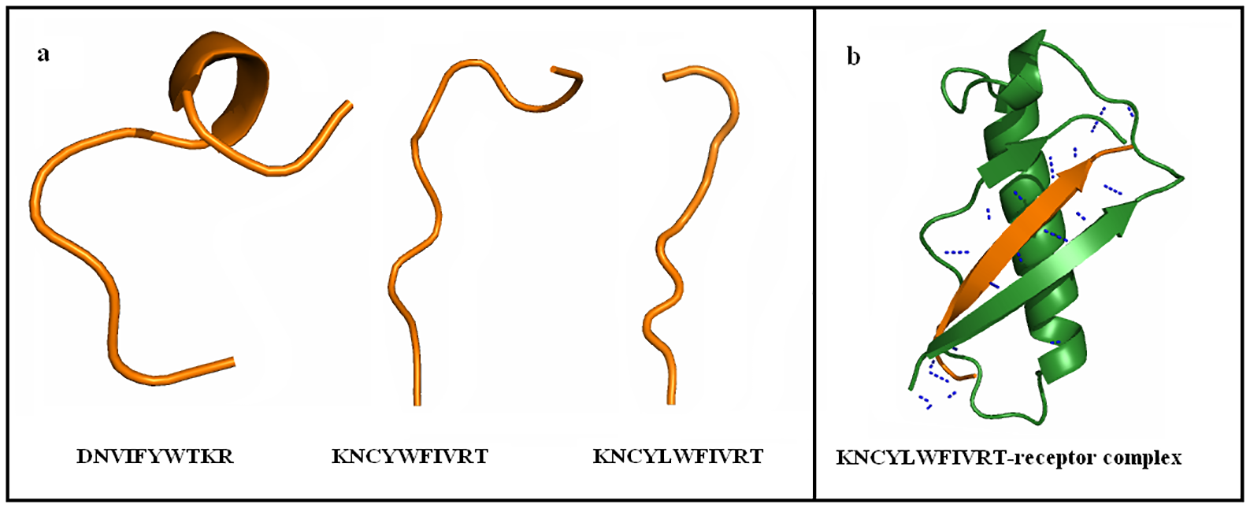
Structure and docking pose of property based peptide (PRP). The figure displays the a) structure of property based peptide and b) binding of PRP at the receptor interface.

Taking into account the linearity of the reference peptide, we opted to design linear peptides. As a result we designed peptides with beta sheet forming amino acids namely cysteine, isoleucine, phenylalanine, threonine, tryptophan and tyrosine [39,40]. Proline and glycine were avoided to reduce folding of the peptide. In order to enable hydrogen bonding we included the charged residues asparagine, aspartic acid, lysine and arginine. This resulted in the peptide sequence DNVIFYWTKR. This peptide however was non-anticancer and therefore mutant peptides with anticancer property and high bioactive scores were selected. Subsequently the peptide KNCYWFIVRT was selected and modeled using PEP-Fold (Fig 3a). The structure was noted to be partially linear and therefore a random insertion mutation was performed to obtain a completely linear peptide sequence KNCYLWFIVRT (Fig 3a). The bioactive and anticancer property was satisfied for the peptide and therefore was subjected to docking and validation. As expected the docking resulted in antiparallel b-sheet hydrogen bonding between the receptor and peptide similar to the reference peptide as shown in Fig 3b. The global energy score of the complex was superior to the reference peptide with a value of -201.1kcal/mol, along with a good hydrogen bond score (Table 7).

**Table 7:** Dock scores of property based peptide.

Peptide	Global Energy	Attractive VdW^a^	Repulsive VdW^a^	ACE^b^	HB^c^
PRP^d^	-202.10	-59.00	8.97	-45.88	-8.12

^a^Vanderwaals,

^b^Atomic Contact Energy,

^c^HydrogenBond,

^d^Property based peptide

### 3.4 Stability checks of docked complexes

**Fig 4:**
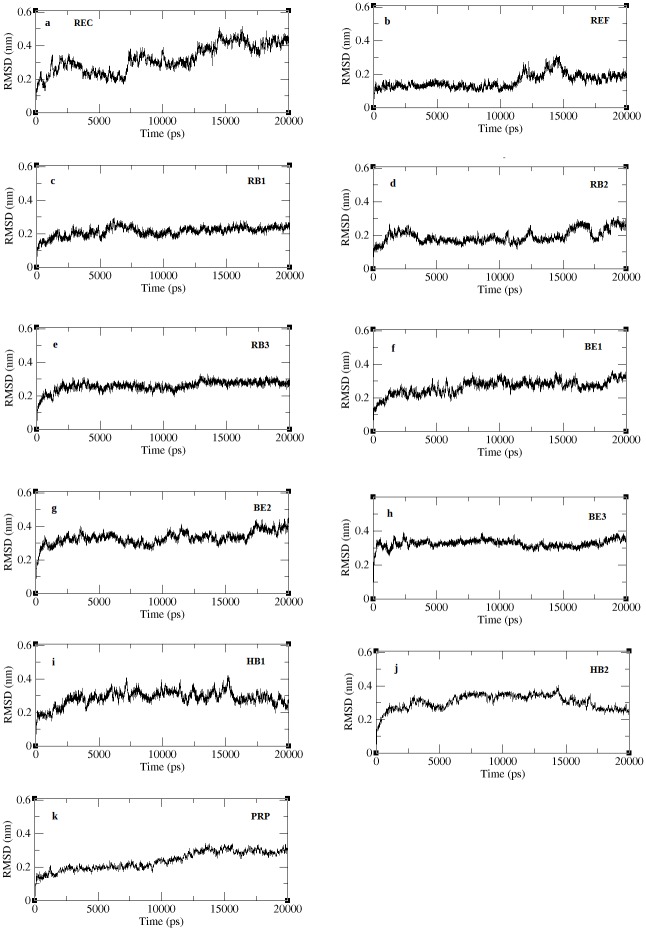
RMSD of peptide bound complexes across the trajectory.

**Fig 5:**
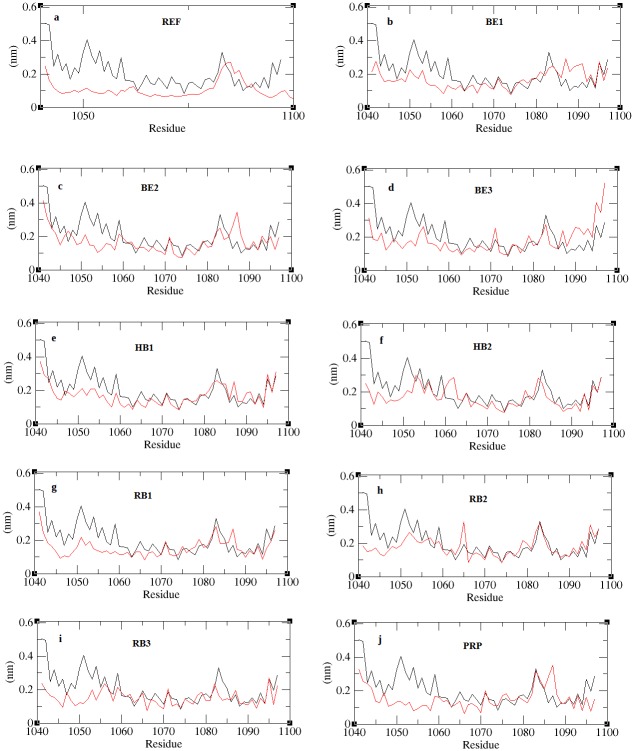
RMSF plots for all peptide bound complexes. The figure shows the residue fluctuations for unbound receptor (black) and peptide bound receptor (red).

The best docking solutions of the protein-peptide complexes validated in firedock were subjected to a 20ns molecular dynamics solution in order to equilibrate interaction regions and obtain a precise structure of the complex close to realistic conditions [41,42]. The complexes included were the REF peptide (SVVVQLTLA) and its derivatives RB1 (SVPLSLCLV), RB2 (SCGLSLCLW) and RB3 (CCVLSLCLV), binding energy based peptides BE1 (GHQWFRFGF), BE2 (PHCWWHWVF) and BE3 (PHCWWLWVF), hydrogen bonds based peptides HB1 (QGHRFWFFG) and HB2 (QPHKFWFFG) and property based peptide, PRP (KNCYLWFIVRT). Stability checks were done by calculating the backbone RMSD of the complexes and the RMSD with respect to the initial structure is plotted in Fig 4. The figure shows all complexes to converge well within few ps of the simulation and the deviations are limited to <0.5nm. The unbound receptor experience structural variations to an RMSD about 0.5nm throughout the entire simulation indicating the instability in its intraprotein interactions. The RMSDs of all the complexes stabilize, although at different time of the simulation. The internal fluctuation of the residues estimated through RMSF was calculated and is reported in Fig 5a–5j. Fluctuations in all the complexes were limited to <0.3nm specifically at the internal residues, which indicates the internal motif, is highly stabilized through the binding of peptides. Among all, the REF peptide bound complex shows a very minimal fluctuation at both the terminal and internal residues confirming a highly stabilized interaction. From the RMSF results we observed that the reference peptide (REF), its second derivatives (RB1 and RB3), and property based peptide (PRP) show stabilized structure of the entire complex including the terminal and internal residues.

#### 3.4.1 Inter protein interactions in complexes

**Fig 6:**
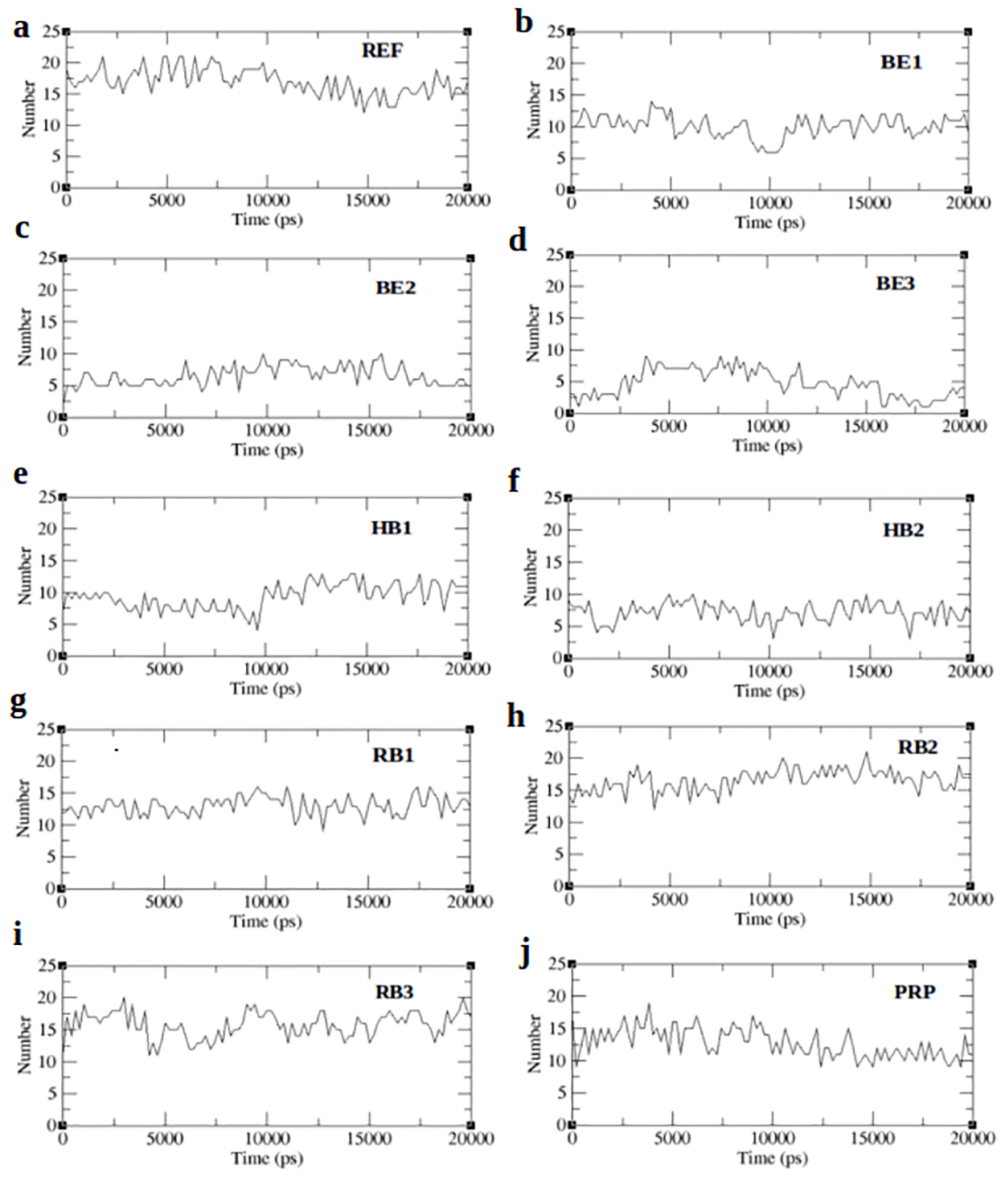
Interprotein hydrogen bonds for peptide bound complexes.

Inter protein interactions predicted across the entire trajectory are reported in terms of the number of hydrogen bonds and is displayed in Fig 6. Strikingly all the complexes except RB2 show lesser number of hydrogen bonds when compared to the receptor peptide, which reaches upto 20 bonds between the receptor and peptide. RB2 peptide shows an increased interprotein hydrogen bonding justifying the RMSF results. From the RMSF results we also expect the RB1 and PRP peptide to show strong hydrogen bonding with the peptide. However in contrast to this we noted lesser bonding in RB1 and PRP complex than the reference. On the other hand, the highest hydrogen bond score predicted by firedock for PEP41 (HB1), shows lesser number of hydrogen bonds. Fig 6 shows that throughout the simulation, the number of hydrogen bonds fluctuates between 1–5, thereby maintaining an average number of bonds between the receptor and peptide. While none of the complexes have inter-protein hydrogen bonds closer to the reference peptide except RB2, it is assumed that the binding affinity of all complexes will be lesser than the reference peptide. In order to confirm this we employed MM-PBSA method to predict the binding affinities of the complexes across the 20ns trajectory.

#### 3.4.2 Binding affinity analysis through MM-PBSA method

MM-PBSA (Molecular Mechanism Poisson–Boltzmann surface area) is a method used to post process docked structures and to evaluate binding free energies of complexes [42, 43]. The binding free energy of the complex is the sum of electrostatic, van der waals, polar and non-polar interactions between the molecules [44, 45]. From each MD-simulated complex, about 300 frames at regular intervals were retrieved from the last 15ns of the trajectory. The binding affinity will thus represent the average of the binding affinities obtained from each of the frames of the specific MD-simulated complex. The results obtained through MM-PBSA calculations are tabulated in Table 8. From Table 8 we observed that the binding energy of RB1 peptide is higher than the reference. This is in par with the RMSF result (Fig 5g), which shows a stable structure of the complex with minimum fluctuation closer to the reference peptide (Fig 5a). Smaller non-polar solvation energy of RB1 further confirms that the complex prefers a compact structure with limited exposure to solvent. Likewise PRP-complex shows a compact structure justifying the RMSF results (Fig 5j). Strikingly the hydrogen bond based peptides (HB1, HB2) show poor ionic interaction and is in par with hydrogen bond results (Fig 6e and 6f) while stronger ionic interactions are observed with the BE1 peptide. All the RB peptides show strong ionic interactions similar to that of the REF peptide. Comparing all the scores we observed that the ionic interactions between the interacting partners should give an estimate of the stability of protein-peptide complex due to its drastic variations in all complexes. The stability of the complexes was further justified by studying the disassociation of the peptide from receptor through steered molecular dynamics (SMD) simulation.

**Table 8:** Binding energies of all peptides.

Si. No.	Peptides	Rank	VdwE^a^	EE^b^	PSA^c^	SASA^d^	BE^e^
1	REF	2	-426.124	-530.982	600.137	-46.304	-403.090
**2**	**RB1**	**1**	**-439.407**	**-540.810**	**615.560**	**-43.788**	**-408.423**
3	RB2	6	-383.782	-518.809	583.981	-43.024	-361.506
4	RB3	4	-432.721	-525.250	608.928	-45.453	-394.525
5	BE1	9	-368.749	-495.913	628.815	-43.732	-279.488
6	BE2	3	-449.302	-360.108	461.182	-47.680	-395.951
7	BE3	8	-448.165	-244.224	441.993	-50.126	-300.652
8	HB1	10	-405.969	-47.956	232.813	-42.193	-263.363
9	HB2	7	-491.747	-160.951	375.673	-47.629	-324.558
10	PRP	5	-500.386	-305.245	496.082	-56.831	-366.182

^a^Vanderwaals Energy,

^b^Electrostatic Energy,

^c^Polar Solvation Energy,

^d^Solvent Accessible Surface Area,

^e^Binding Energy.

Top scoring peptide is highlighted in bold

### 3.5 Steered molecular dynamics simulation

SMD simulation was performed on the selected REF, RB, BE, HB and PRP peptide complexes, to validate the binding energies estimated across the trajectory frames. SMD is an approach analogous to atomic force microscope [33, 41] in which, an external force is applied to the ligand to pull it away from the protein. It can be inferred that the magnitude of the pulling force required to completely dissociate the peptide from the receptor is directly proportional to the binding affinity of the peptide.

#### 3.5.1 Determination of the pulling velocity and spring constant

Spring forces are dependent on the pulling velocity and spring constant applied on a system. Smaller pulling velocity and a larger spring constant is usually preferred to avoid any dis-equilibration of the system and to achieve a measurable disassociation potential respectively. Therefore prior to applying an external harmonic potential, it is mandatory to evaluate the choices of spring constants and velocities for our system [46]. Different parameters such as spring constant (k) and velocity (v) were initially tested to identify the optimal combinations for the present SMD simulations. Pulling simulations at different velocities ranging from 0.0008 nm/ps to 0.008 nm/ps were tested on the reference peptide using a constant force with simulation time ranging from 1ns to 15ns. Triplicates were done for each of the velocities. Eventually the rupture forces for every pulling velocity were identified and are plotted in S3a Fig. There was a linear correlation between rupture force and pulling velocity for velocities above 0.0008nm/ps. Therefore, for all the SMD simulations 0.0008nm/ps were chosen as a constant velocity. Similarly 8 spring constants were evaluated viz 100, 300, 600, 800, 1000, 2000, 3000 and 4000 kJ/mol/nm^2^. On comparing the rupture forces from all the spring constants as shown in S3b Fig, we noted that lower spring constants (100–1000 kJ/mol/nm^2^) had no effect on the structure. Spring constants 3000 kJ/mol/nm^2^ and 4000 kJ/mol/nm^2^ induced larger fluctuations while at 2000 kJ/mol/nm2 the force induced gradual and milder fluctuations which will be useful to evaluate the unbinding process. Apparently a spring constant of 2000 kJ/mol/nm^2^ was chosen for all pulling simulations.

#### 3.5.2 Pulling simulations in the protein-peptide complexes

**Fig 7:**
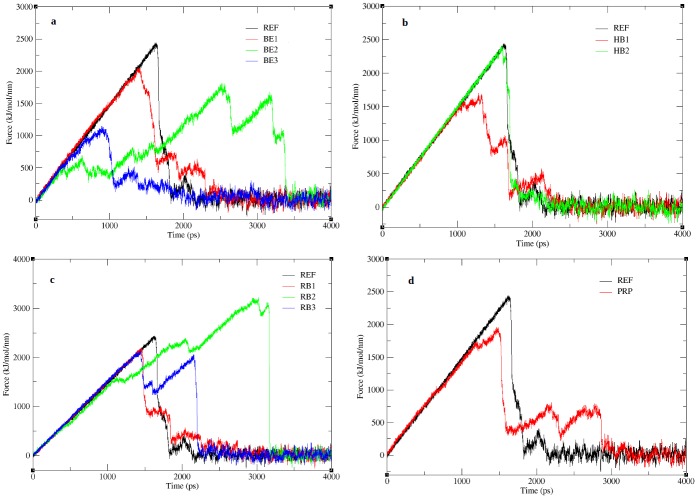
Pull force applied on peptide during SMD. The figure shows the time dependent external force applied to pull a) binding energy based peptides, b) hydrogen bond based peptides, c) reference based peptides and d) property based peptide.

SMD simulation was performed for 4ns for each complex to allow the complete unbinding of the peptide. Pulling force applied to the BE peptides, show the peptides (BE1, BE2, BE3) to disassociate from the receptor much earlier than the REF peptide (Fig 7a). The unbinding frames for all peptides complexes obtained from the pulling simulations are shown in S4 Fig. Snapshots of BE1 peptide disassociation (S4a Fig) clearly displays a sequential unbinding process which completes quickly. Contrarily, for BE2 (S4b Fig) the unbinding starts very early, however the cleavage of stronger bonds towards the N-terminal delays the unbinding process as a result requires a higher force. In BE3 (S4c Fig), the binding is short lived as only the internal residues are involved in interaction with the receptor, therefore requires a very small force. While HB1 disassociates much faster (Fig 7b), the HB2 peptide stays in the bonded state for a longer time, indicating the strength of the hydrogen bonds, nevertheless do not exceed the REF peptide. The unbinding process in the HB1 (S4d Fig) initiates from the C-terminus while in HB2 (S4e Fig) it starts from the N-terminus, which indicates that weakly bound residues are susceptible to external forces. However the RB peptides show better results than the BE and HB peptides in both binding energy and pulling, indicating the receptor’s preference for a native peptide (Fig 7c). RB1 though is closer to the receptor, the pull force do not exceed the receptor (Fig 7c), which is in acceptance with the binding scores. The snapshots from the RB1 (S4g Fig) pulling trajectory show the external force affects all the peptide residues simultaneously allowing the complete unbinding of the peptide. Among the three RB peptides only RB2 appear to exceed the pull force which is strengthened by the electrostatic interaction (Table 8). Unlike RB1 peptide, disassociation of RB2 peptide occurs sequentially (S4h Fig), requiring an increase in force to unbind residues as the simulation proceeds. RB3 peptide disassociation (S4i Fig) also proceeds gradually initiating from the N-terminus, and requires a higher force to unbind successive residues. Similar to BE peptides, the PRP peptide also show a lesser pull force (Fig 7d), indicating a poor binding affinity, due to lesser intramolecular hydrogen bonds as inferred from the electrostatic interaction score (Table 8). Though we observe antiparallel beta sheet hydrogen bonding in PRP peptide, lesser force was used since the unbinding initiated from the internal residues as shown in S4f Fig at 1000ps. Therefore the bonds at terminal ends were destabilized and were disassociated earlier without requiring much force. In the REF peptide the disassociation appear to be sequential (S4j Fig) and proceeds from the C-terminal. The pulling simulations show that the time for the peptide to unbind from the protein in all complexes vary between 1000-3500ps (Fig 7) and the time for the complete disassociation of peptide in all complexes is given in Table 9. Though the peptides BE2, RB2, and PRP requires a longer time for disassociation, from Fig 7 we observed that when the peptides starts disassociating earlier, it partially unbinds and then requires a larger force to completely disassociate. This delay is attributed to the strength of specific bonds between the peptide and protein which requires slightly larger forces than that of the initial unbinding. To identify such bonds screen shots of protein-peptide complexes were taken at random time intervals between the time of the start and end of peptide disassociation. From each frame, the residue that bonds with the receptor until the time of complete disassociation was identified. For instance, in the REF peptide, the pulling starts at the 6^th^ position, followed by the residue at 5^th^ position. Here, we reason that the last residue to be unbonded will be the residue that has higher bond strength. In case of the REF peptide, the residue SER at 1^st^ position is the last to be unbonded and hence has stronger bond strength. In this view, all residues that require high pull energy in each of the complexes were predicted and are tabulated in S5 Table. From the list of residues in S5 Table, we selected for each position among all complexes, the residues that would last longer during the simulation in a bonded conformation and the consolidated list is given in Table 10. The residues in the Table 10 thus represent the choice of its position in a 9-mer peptide sequence to be tightly bonded with the receptor. A peptide derived from this table is thus expected to poses a higher binding affinity with increased pull force to be disassociated from the receptor.

**Table 9:** Time for complete disassociation of peptides.

Peptides	Start time of disassociation (ps)	End of disassociation (ps)	Total time for disassociation (ps)
REF	1320	2600	1280
BE1	1550	2350	800
BE2	450	3340	2890
BE3	920	1650	730
HB1	1230	2200	970
HB2	1620	1900	280
RB1	1460	2690	1230
RB2	1700	2860	1160
RB3	1600	2050	450
PRP	1220	2800	1580

**Table 10:** Consolidated list of residues requiring high pull force.

Positions	Residues		
1	PRO		
2	HIS		
3	CYS		
4	LEU		
5	SER		
6	LEU	TRP	
7	CYS		
8	PHE	LEU	
9	TRP	GLY	VAL

### 3.6 Modeling, docking and simulation of pull based peptide (PF)

**Fig 8:**
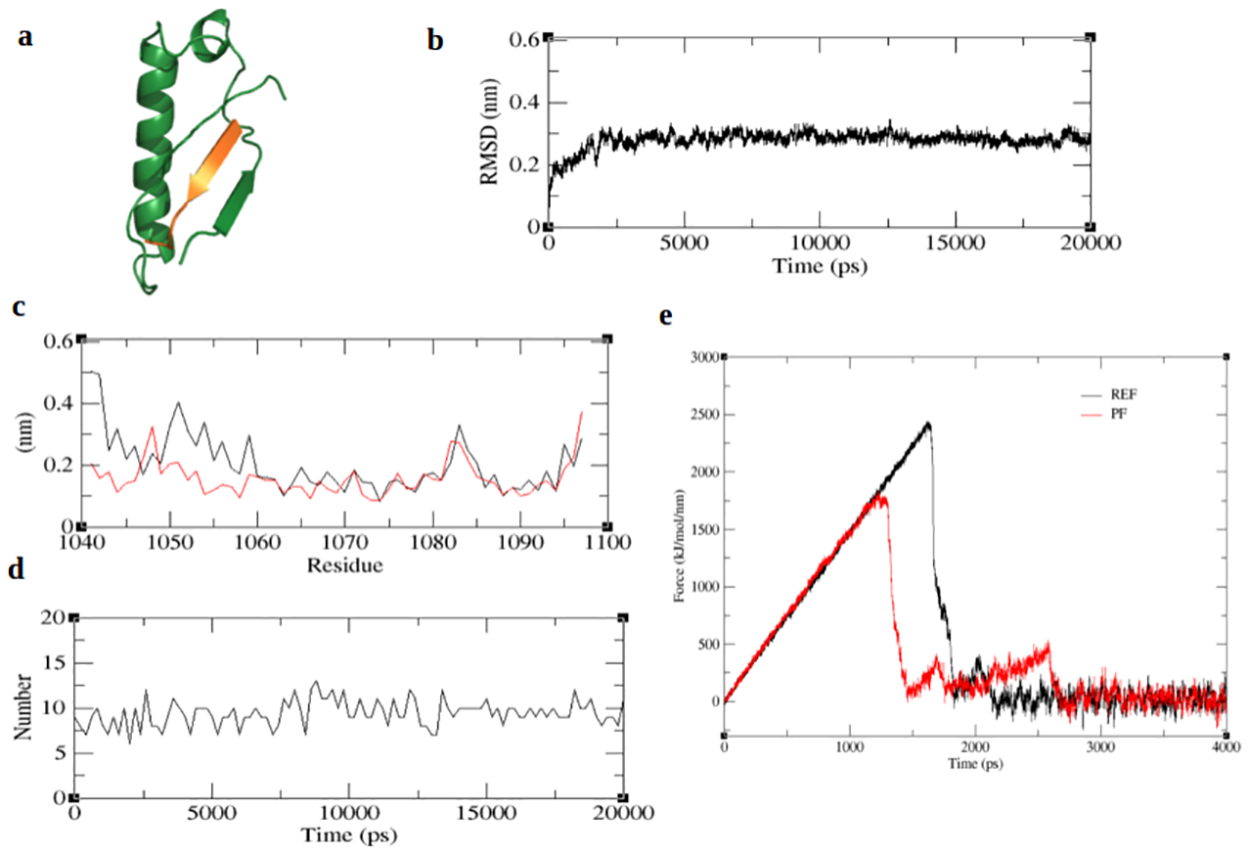
Binding affinity and simulation of pull based peptide (PF). The figure represents a) docked structure of PF, b) backbone RMSD of PF-complex, c) RMSF of PF (red) in comparison with the unbound receptor (black), d) number of inter-protein hydrogen bonds in PF-complex and e) PF disassociation (red) in comparison with the REF peptide (black).

From Table 10, we derived several peptides. However, taking into account, the anticp, bioactivity scores and linear conformation of the peptide, we obtained a 9-mer peptide sequence viz PHCLSWCFW to have good bioactive scores, anticp property and a linear conformation from Peptide Ranker, AntiCP and PEP-FOLD programs respectively. The modeled peptide was then subjected to docking and refinement similar to other peptides. The binding affinity of the peptide obtained from firedock is given in Table 11a and the docked structure is given in Fig 8a. Unfortunately, the global energy was noted to be much lesser compared to the reference peptide. Fig 8a shows an antiparallel β sheet formation between the peptide and receptor, which indicate favorably bonded interaction. The pull force based peptide complex (PF) was subjected to simulation similar to the other peptides. The RMSD plot (Fig 8b) obtained from the 20ns trajectory shows the values to converge within 0.4nm indicating the stability of the complex. Fluctuation of residues obtained from RMSF analysis (Fig 8c) show all N-terminal residues to exhibit only minor fluctuations indicating strong association of the N-terminal residues with the receptor. Unlike the RB and PRP peptides the inter protein hydrogen bonds of PF complex was limited to <10 bonds (Fig 8d). The binding energy of PF peptide was calculated using MM-PBSA method is reported in Table 11b. Unexpectedly the binding energy of PF peptide complex was much lower than the REF peptide and all other peptide complexes. Eventually, we predicted the extent of peptide affinity by subjecting the PF complex to a 4ns SMD simulation similar to other peptides. The time for the complete disassociation of peptide was identified from the SMD trajectory and is given in Table 11c. The peptide starts to unbind earlier than the reference peptide and also takes about 2000ps for the complete disassociation of the peptide (Fig 8e). As observed from the figure (Fig 8e) only the terminal end of the peptide continues in the bound state as a result the disassociation time is delayed. Additively when compared to all protein-peptide complexes RB2 shows the highest binding affinity. The maximum pull force required to pull the peptide exceeds the REF peptide as shown in S5 Fig.

**Table 11:** Binding energy values of pull based peptide (PF).

a	**Peptide**	**Global energy**	**Attractive VdW**^a^	**Repulsive VdW**^a^	**ACE**^b^	**HB**^c^
	Pf	-158.5	-49.08	4.78	-45.76	-3.96
b	**Peptide**	**VdwE**^d^	**EE**^e^	**PSA**^f^	**SASA**^g^	**BE**^h^
	Pf	-465.486	-53.803	290.734	-50.036	-278.598
c	**Peptide**	**Start time of disassociation (ps)**		**End of disassociation (ps)**		**Total time for disassociation (ps)**
	Pf	1250		3250		2000

^a^Vanderwaals,

^b^Atomic Contact Energy,

^c^HydrogenBond,

^d^Vanderwaals Energy,

^e^Electrostatic Energy,

^f^Polar Solvation Energy,

^g^Solvent Accessible Surface Area,

^h^Binding Energy.

### 3.7 Umbrella sampling and PMF calculation

**Fig 9:**
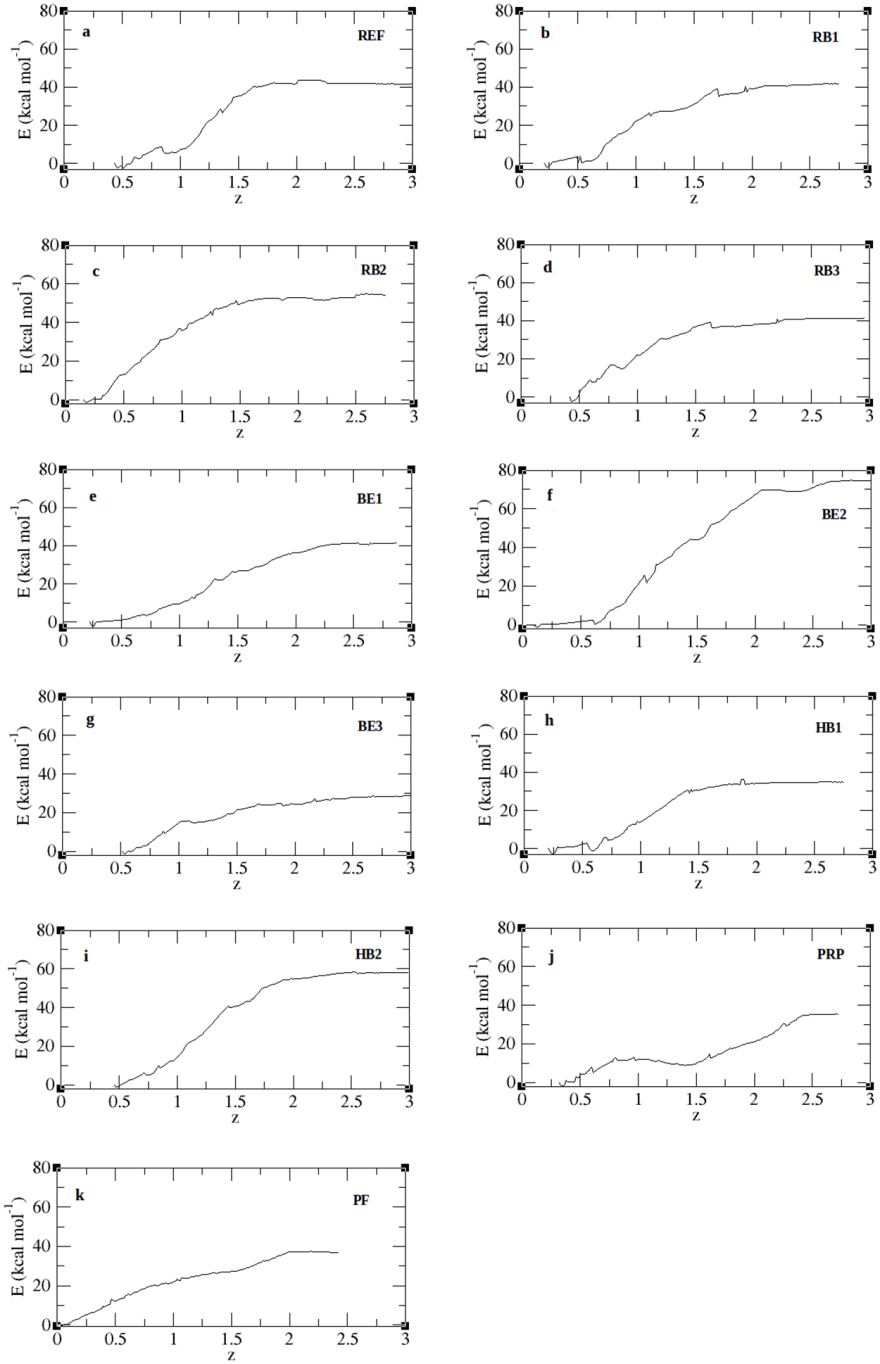
PMF plot for protein-peptide complexes. Figure denotes the potential of mean force (y-axis) acting on the spring to disassociate the peptide across the reaction coordinate (x-axis).

As an extension of the SMD analysis, we calculated the PMF profiles of peptide unbinding through umbrella sampling. Fig 9 shows the PMF profiles for all peptide complexes along the reaction coordinate covering a distance from 0.2nm– 3.0nm and the individual free energies calculated from the PMF plot are given in Table 12. From Fig 9 we noted that all complexes attain a plateau with an unbinding free energy (δG) ranging between 28-73kcal/mol. Though the REF peptide (Fig 9a) and RB1 (Fig 9b) are close enough they take a different path in the initial phase, however they reach the same plateau. The lower PMF values of the peptides BE1 (Fig 9e), BE3 (Fig 9g), HB1 (Fig 9h), PRP (Fig 9j) and PF (Fig 9k)) show that they can easily unbind from the protein. On the other hand, BE2 (Fig 9f) peptide shows the highest free energy of 73.83kcal/mol followed by HB2 (Fig 9i) and RB2 (Fig 9c) with 58.06kcal/mol and 54.83kcal/mol respectively. These peptides therefore need to overcome a high energy barrier in its unbinding process along the reaction coordinate. This high energy barrier is an indicative of a strong protein-peptide interaction [36]. To understand this, the binding energy contributions of individual residues from all peptide complexes were predicted and listed in S6 Table and their overall binding energy scores are given in Table 12. We noted that for BE2 the residues PRO1 and TRP5 together extend a high binding energy of -83.56kJ/mol. Similarly the strong energy barrier for RB2 was noted to be due to the residues LEU4 and LEU6 and in HB2 the residueTRP6. In BE1 and BE3 only one among the 9 residues contributed to strong binding. Similarly in HB1 the terminal residues contributed more towards the binding energy. Despite having more number of residues, the PRP peptide binding is supported only by ARG10 solely contributing to energy of -58kJ/mol towards the overall residue binding energy. On the contrary, the PF peptide derived from the pulling forces of individual residues, shows a very poor affinity which is inferred from both the unbinding potential and residue energy contribution [Table 12]. According to the PMF profiles the peptides BE2, HB2 and RB2 show a stronger binding affinity towards the receptor protein. We noted that for BE2 the residues PRO1 and TRP5 together extend a high binding energy of -83.56kJ/mol. Similarly the strong energy barrier for RB2 was noted to be due to the residues LEU4 and LEU6 and in HB2 the residueTRP6. In BE1 and BE3 only one among the 9 residues contributed to strong binding. Similarly in HB1 the terminal residues contributed more towards the binding energy. Despite having more number of residues, the PRP peptide binding is supported only by ARG10 solely contributing to energy of -58kJ/mol towards the overall residue binding energy. On the contrary, the PF peptide derived from the pulling forces of individual residues, shows a very poor affinity which in inferred from both the unbinding potential and residue energy contribution. According to the PMF profiles the peptides BE2, HB2 and RB2 show a stronger binding affinity towards the receptor protein.

**Table 12:** Unbinding free energies (δG) and residue contribution towards interprotein interactions.

Si. No.	Peptides	δGbind (kCal/mol)	Total residue binding energy (kJ/mol)	Rank by PMF
1	REF	41.50	-224.78	4
2	BE1	40.98	-152.27	6
3	BE2	73.83	-190.36	1
4	BE3	28.40	-162.60	11
5	HB1	34.20	-168.81	10
6	HB2	58.06	-151.78	2
**7**	**RB1**	**41.45**	**-229.44**	**5**
8	RB2	54.83	-180.94	3
9	RB3	40.72	-212.97	7
10	PRP	34.22	-218.21	9
11	PF	37.20	-148.69	8

Top scoring peptide is highlighted in bold

## 4. Discussion

Mucin 1 is a widely used prognostic breast cancer tumor marker. The functional form of mucin 1 is formed by an auto cleavage process resulting in 2 subunits designated α and β. The subunits reassociate to form the functional heterodimeric structure of mucin 1. The alpha subunit is cytosolic and beta subunit is associated with the transmembrane segment and therefore is membrane bound. This subunit reassociation is proposed to play a key role in disease progression primarily in breast cancers. Therefore inhibition of this association is anticipated to provide a therapeutical effect in carcinogenesis. To address this issue, we computationally evaluated several peptide ligands against the subunit interface through docking and simulation studies. Since the interacting partners are protein units, peptide ligands were preferred as source of subunit inhibition. The cytosolic subunit was treated as the receptor. Several peptides were computationally designed using various approaches. Derivative peptides were designed by generating mutant and mutant combinations of the candidate peptides. Anticancer property and bioactivity were used as validation criteria for all the peptides used in this study, in order to obtain a peptide that might be functionally active.

Subunit reassociation in MUC1 is reported to be highly specific, in which the receptor provides the key residues for its ligand, and thereby prevents any non-specific interactions. In view of this, the use of a reference peptide sequence, as a direct inhibitor would be meaningful, nevertheless the reference peptide represented by a 9 residue sequence “SVVVQLTLA”, was reported to be non-anticancer peptide and non-bioactive. Docking of the reference peptide derivatives even upto the 3^rd^ generation resulted in a dock score much lower than the reference peptide. This implies that modification or mutation of the reference peptides reduces its binding affinity and therefore further alterations on the reference peptide derivatives were not performed. This necessitated the need for designing novel peptides that can provide a competitive inhibition of the subunit reassociation. Eventually the interface residues involved in the subunit interactions were chosen for peptide designing. Since the native subunit interaction forms an anti-parallel β-sheet formation, a 9-mer peptide length was considered optimal, to avoid intra-peptide folding. Initiating with the “GFHNFRQFG” sequence, peptide derivatives were obtained and docking solutions were acquired similar to reference peptide. Additively, we had also taken into consideration, the occupancy of peptide at the binding site.

Among the 49 peptides from the initial library only the peptide “GHQWFRFGF” was noted to completely occupy the subunit interface. As a result subsequent mutations were generated and its derivatives were obtained upto the 4^th^ generation through combinatorial mutations. Nevertheless, best global energy was obtained from the third generation peptides, with “PHCWWLWVF” being the best docked solution. Therefore the third generation peptides were considered as maximally optimal from the binding energy based peptides (BE). Despite the good binding energy scores of the BE peptides, the binding scores were largely contributed by van der waals force and resulted in lesser hydrogen bond score. Since hydrogen bonds contribute largely to the stability of the docked complex, peptides with high hydrogen bond score (HB) were selected from the initial library and tested for their binding efficacy at the interface. Unfortunately, mutations of HB peptides resulted in low hydrogen bond score with global energy scores lesser than the reference peptide. From the BE and HB peptide docking studies, we observed that, when the peptides bound in a linear conformation they tend to form antiparallel beta sheet with the subunit similar to the reference peptide. Strikingly we noted that all peptides from initial library tend to coil or randomly fold while interacting with the subunit or only partly form beta sheet conformations. Therefore in order to predict if beta sheet conformation could enhance the binding affinity of the peptide we designed linear peptides. The results obtained from such property based peptides (PRP) or linear peptides were satisfying in both global energy and hydrogen bond score. We could also visualize the native antiparallel beta sheet interactions with the complete occupancy of the peptide at the subunit interface.

The best docked complexes namely REF, RB, BE, HB and PRP bound complexes were evaluated for their stability which revealed that all bound complexes were stable than the unbound receptor. Minimal deviation in the RMSD of all complexes states that the peptide binding induces the stable conformation of the receptor. Further, residue fluctuations in all bound complexes when compared with the unbound receptor were noted to be lesser indicating stability of residue conformation within the structure. In addition, the terminal residues in all complexes exhibited a lesser fluctuations owing to their interactions with the peptide. Inter protein interactions that relate to the stability of all the peptide docked complexes reported that in comparison to all complexes the receptor based derivatives (RB2 and RB3) have the maximum number of hydrogen bonds other than the reference peptide. However, we noted that the dock scores obtained from firedock, did not correlate to the hydrogen bonds predicted across the trajectory. This contradiction in dock scores and trajectory analysis was reasoned to be due to the scores obtained from static models of the receptor-peptide complexes.

While simulating the complex in a solvent environment, the number of hydrogen bonds tends to fluctuate which are due to the formation of newer hydrogen bonds or breaking of existing bonds as simulation proceeds. However, the persistence of the existing hydrogen bonds indicates the stability of the inter peptide interactions. As a result, the persistence or stability of the hydrogen bonds across the simulation was estimated using MM-PBSA method. As expected, excepting the receptor based peptide derivative (RB1), the binding energies of all the complexes were lesser compared to the reference peptide. The reason being that RB1 peptide was equally stabilized by van der Waals and electrostatic interactions, while the reference peptide was stabilized by electrostatic interaction. Additively, the PRP-complex was also stabilized by van der waals force, nevertheless the binding affinity was comparatively lower than reference peptide due to a low electrostatic interaction. The stronger ionic interaction observed in the binding energy based peptide (BE1) and a reduced score in hydrogen bond based peptide (HB1 and HB2) complexes indicate the reduced preference for foreign residues at its interface. On comparing the individual scores, we observed that all the peptide complexes are stabilized through van der Waals interaction, nevertheless drastic variations were observed with the ionic interactions scores. This postulates that the binding affinity of the peptides, according to the present study, is dependent on the electrostatic interaction between the peptide and protein.

Peptides inhibiting the subunit reassociation of the MUC1 heterodimers, should work as a competitive inhibitor against the ligand subunit preventing its interaction with the receptor. Binding affinity analysis reported the receptor based (RB1) peptide to have a good binding affinity closer to the reference peptide. However, in presence of the ligand subunit, *in vivo*, the affinity of the inhibitor peptide needs to be evaluated. Eventually SMD simulations were performed and the results reported that the pull force of BE and HB peptides were contradicting with the binding energy scores. Where, a higher pull force was associated with lower binding energy and vice versa. This contradiction was attributed to the strength of the bonds extended by, not all, but fewer peptide residues thereby reducing the average binding energy of the peptide and increasing the pull force to cleave the strongly bonded residue(s). As observed from the trajectory analysis and binding affinity through MM-PBSA method, the RB2 and HB2 peptides require a large force to disassociate from the protein. Compared to the REF peptide, RB2 peptide starts to disassociate at a later phase indicating its strength of binding. The delay in time to unbind peptide also indicates an increase in the pull force to pull the peptide away from the receptor.

Since the pull force is directly proportional to strength of hydrogen bond, the residue that is pulled away finally will be the residue that requires a larger force. Likewise the residue to be unbonded initially during the pull simulation would require smaller force and hence get cleaved. Eventually from all the complexes we identified for each position the residue that is finally pulled irrespective of the peptide sequence. The interaction patterns obtained from all the peptide complexes paved way for designing pull based peptide (PF). With the prediction of residues that gets last unbonded from the receptor; we designed a new peptide which was expected to exhibit higher binding affinity with the receptor. Unexpectedly the quantitative measures of docking and simulation of PF peptide was lower than all the peptide complexes and was noted to be coherent in both binding energy and pull force. The PF peptide complex was stabilized by vanderwaals interaction and had a very low electrostatic interaction which indicates a poor bond strength justified by lesser number of hydrogen bonds between the peptide and receptor. The weaker interactions require smaller forces for unbinding and are well evident from PF peptide disassociation. Free energy potentials predicted from umbrella sampling of SMD configurations indicated a strong correlation between individual residue contribution and PMF profiles. While binding energy, pulling forces and free energy barrier of disassociation were completely contradictory, the contribution of individual residues towards interprotein interactions clearly depicted its correlation. Disassociation of peptide initiating from N-terminal end required a larger force and also delayed the unbinding, while it was vice versa when initiated from C-terminal. This indicates residues towards the N-terminus are more vital in contributing towards the inter-protein interactions. Also when continuous stretch of residues shows a stronger bonded interaction, the unbinding was comparatively faster since the force initially acting on the residue was sufficient to pull away the peptide. As a result the peptides BE2, HB2 and RB2 each having two residues contributing a high binding energy in the inter-protein interaction, requires a larger force to act on these residues and therefore retain in the bonded conformation for a longer time. Bond strengthening at the terminal ends accompanied by a stronger bond with an internal residue required a larger force to act. Though anti-parallel beta sheet interaction between protein-peptide complex proved beneficial for a stronger binding energy, the secondary structure has no distinct effect on the pulling simulations and free energy barrier calculation, since a random interaction and anti-parallel beta sheet interactions gave similar results. Also the length of the peptide had a contradictory effect, since the longer PRP peptide was pulled away much earlier contributing to a lesser force and work. Lower PMF value of the PF peptide states that, it is not the mere position of residues, but also the neighboring residue that play a vital role in strengthening the bonds of protein-peptide interaction. Despite several inferences made from the docking, simulation, SMD and PMF calculations, it was noted that the protein-peptide interactions was not specific towards the length, secondary structure, binding energy or hydrogen bonds. A comparison of all the results obtained from docking to PMF calculation, the RB1 peptide gave consistent results showing a closer resemblance towards the REF peptide in binding energy, force, residue contribution and unbinding free energy. Therefore by comparing all the peptide complexes we were able to conclude that only RB1 peptide provides a favorable interaction and further justifies the specificity of receptor-ligand alliance of mucin 1. Nevertheless inferences made in this study serve as criteria for designing and validating peptide inhibitors against mucin 1.

## Supporting information

S1 Fig 3-dimensional structures of interface based peptide. **Color**represent helix (blue), sheets (red), turn (green) and coils (grey)
(TIF)

S2 Fig Docking poses of peptides with high hydrogen bond scores. The figure shows the interface regions highlighted in dark grey and the peptides in blue.
(TIF)

S3 Fig Determination of pulling speed and spring constant. Figure showsa) the rupture forces predicted as a function of pulling velocity and b) the variousspring constants applied to pull the peptide.
(TIF)

S4 Fig Steered molecular dynamics simulation of protein-peptide complexes. The figure shows the time evolution of rupture forces required to disassociate the peptides.
(TIF)

S5 Fig Pull force applied on all peptides during SMD. (TIF)

S1 Table RMSD of NMR conformations of MUC1 SEA domain. (DOCX)

S2 Table Aminoacid substitutions table. Letters represent the single letter codes of amino acids.
(DOCX)

S3 Table Interacting residues between the heterodimeric subunits of MUC1 SEA domain. (DOCX)

S4 Table Top scoring peptide solutions of PEP41 mutants. (DOCX)

S5 Table Peptide residues disassociation patterns. (DOCX)

S6 Table Peptide residue contribution towards protein-peptide interaction. (DOCX)
