# Supplementary figures and images for: Evaluation of peptide designing strategy against subunit reassociation in mucin 1: A steered molecular dynamics approach
Source: PLoS One. 2017 Aug 17;12(8):e0183041. doi: 10.1371/journal.pone.0183041 (PMC5560680; doi:10.1371/journal.pone.0183041)

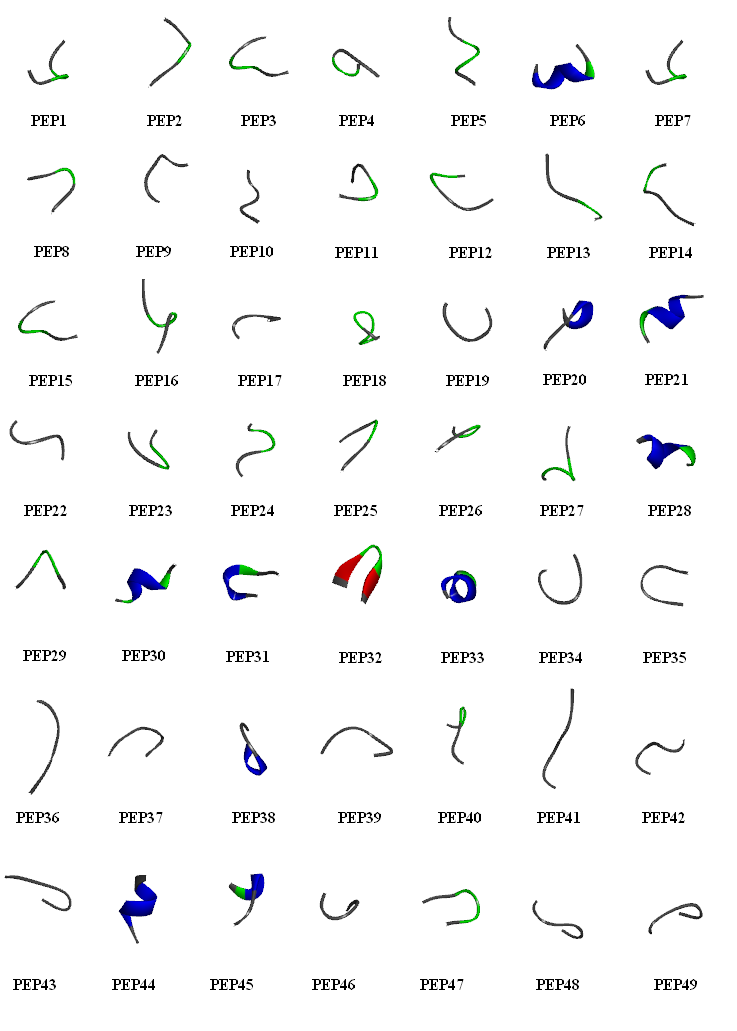

Supplement: S1 Fig — Colorrepresent helix (blue), sheets (red), turn (green) and coils (grey) (TIF) [file pone.0183041.s001.TIF]

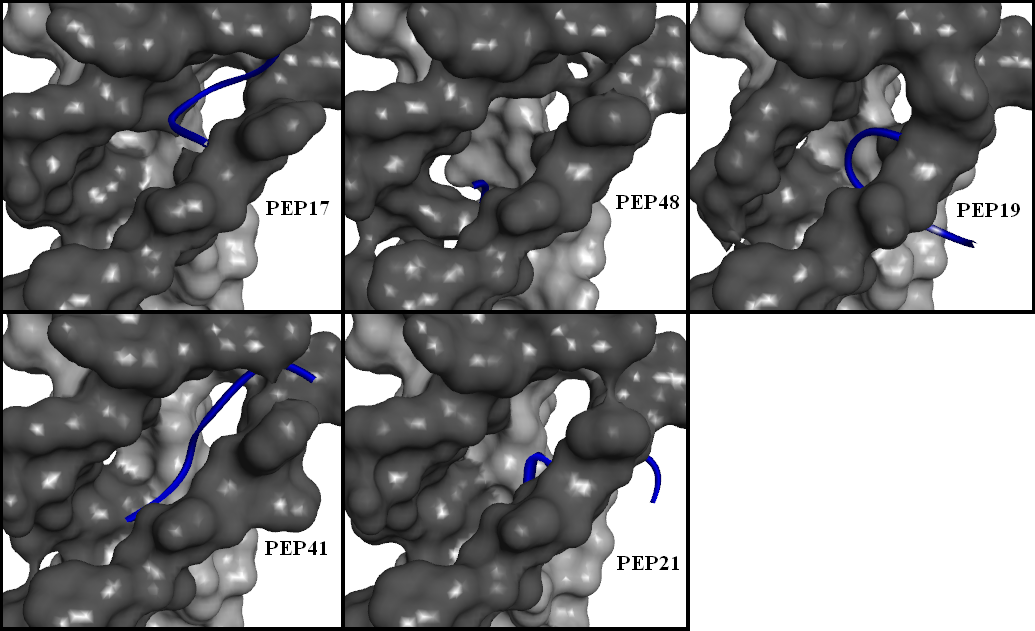

Supplement: S2 Fig — The figure shows the interface regions highlighted in dark grey and the peptides in blue. (TIF) [file pone.0183041.s002.TIF]

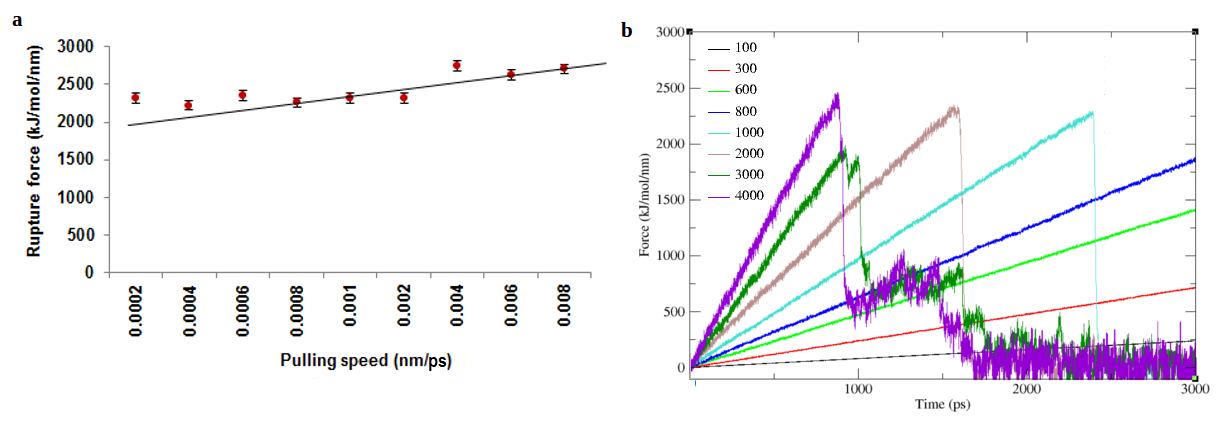

Supplement: S3 Fig — Figure showsa) the rupture forces predicted as a function of pulling velocity and b) the variousspring constants applied to pull the peptide. (TIF) [file pone.0183041.s003.tif]

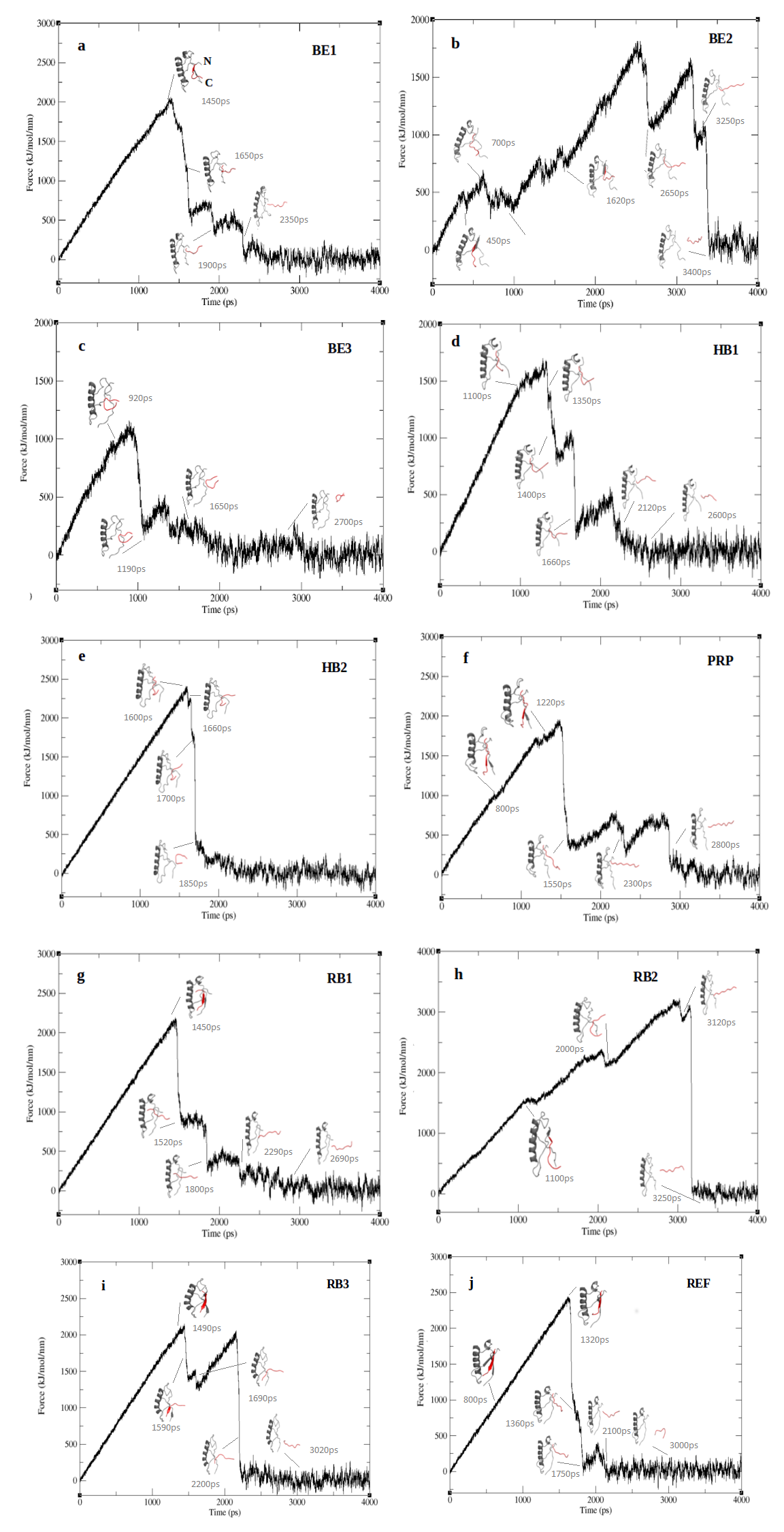

Supplement: S4 Fig — The figure shows the time evolution of rupture forces required to disassociate the peptides. (TIF) [file pone.0183041.s004.TIF]

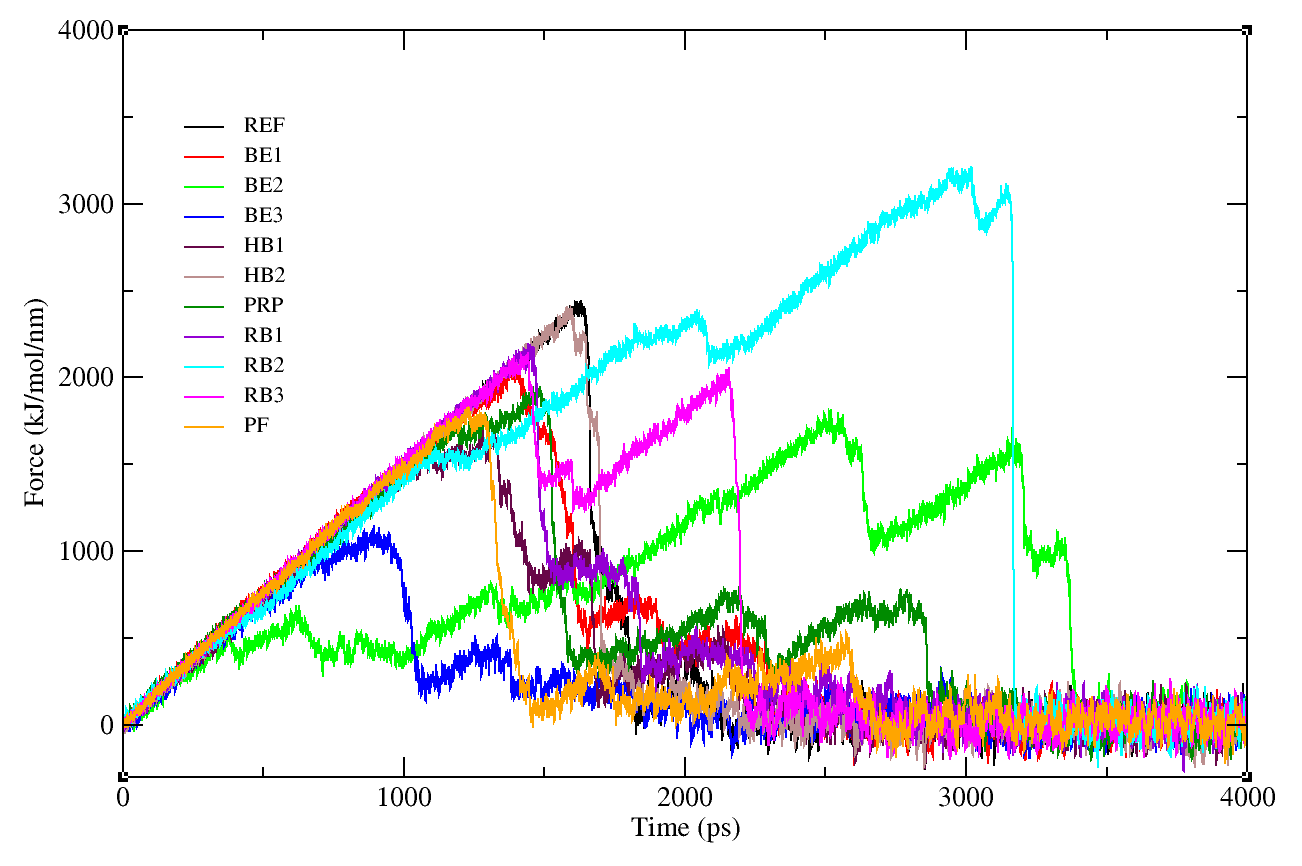

Supplement: S5 Fig — (TIF) [file pone.0183041.s005.TIF]
