# Supplementary material for: Evaluation of peptide designing strategy against subunit reassociation in mucin 1: A steered molecular dynamics approach
Source: PLoS One. 2017 Aug 17;12(8):e0183041. doi: 10.1371/journal.pone.0183041 (PMC5560680; doi:10.1371/journal.pone.0183041)
Supplement: S1 Table — (DOCX) [file pone.0183041.s006.docx]

**S1 Table. RMSD of NMR conformations of MUC1 SEA domain**

| **Models** | **Avg_Rmsd** | **Most deviating structures** | **Large RMSD** | **Identical structures** |
| --- | --- | --- | --- | --- |
| Model 1 | 0.95 | model 12 | 1.247 | model 6 |
| Model 2 | 1.1 | model 11 | 1.266 | nil |
| Model 3 | 1.04 | model 12 | 1.242 | model 7 |
| Model 4 | 1 | model 5,13 | 1.195 | model 10 |
| Model 5 | 1.07 | model 12 | 1.313 | model 13 |
| Model 6 | 0.95 | model 12 | 1.247 | model 1 |
| Model 7 | 1.04 | model 12 | 1.242 | model 3 |
| **Model 8** | **0.94** | **model 12** | **1.18** | **model 14** |
| Model 9 | 1 | model 11 | 1.178 | model 15 |
| Model 10 | 1 | model 5,13 | 1.195 | model 4 |
| Model 11 | 1.18 | model 5,13 | 1.311 | nil |
| Model 12 | 1.21 | model 5,13 | 1.313 | nil |
| Model 13 | 1.07 | model 12 | 1.313 | model 5 |
| **Model 14** | **0.94** | **model 12** | **1.18** | **model 8** |
| Model 15 | 1 | model 11 | 1.178 | model 9 |

Models with lowest RMSD’s are highlighted in bold
