# Supplementary material for: Evaluation of peptide designing strategy against subunit reassociation in mucin 1: A steered molecular dynamics approach
Source: PLoS One. 2017 Aug 17;12(8):e0183041. doi: 10.1371/journal.pone.0183041 (PMC5560680; doi:10.1371/journal.pone.0183041)
Supplement: S2 Table — Letters represent the single letter codes of amino acids. (DOCX) [file pone.0183041.s007.docx]

**S2 Table. Aminoacid substitutions table**

| **R** | **G** | **T** | **F** | **E** |
| --- | --- | --- | --- | --- |
| **SUBSTITUTIONS** | | | | |
| H | A | S | Y | D |
| K | V | C | W | - |
| - | P | N | - | - |
| - | L | Q | - | - |
| - | I | - | - | - |

Letters represent the single letter codes of amino acids
