# Supplementary material for: Evaluation of peptide designing strategy against subunit reassociation in mucin 1: A steered molecular dynamics approach
Source: PLoS One. 2017 Aug 17;12(8):e0183041. doi: 10.1371/journal.pone.0183041 (PMC5560680; doi:10.1371/journal.pone.0183041)
Supplement: S3 Table — (DOCX) [file pone.0183041.s008.docx]

**S3 Table. Interacting residues between the heterodimeric subunits of MUC1 SEA domain**

| **Chain** | **Residue** | **Position** | **Chain** | **Residue** | **Position** |
| --- | --- | --- | --- | --- | --- |
| A | SER | 1041 | B | PHE | 1107 |
| A | PHE | 1043 | B | LEU | 1105 |
| A | PHE | 1044 | B | SER | 1142 |
| A | LEU | 1045 | B | LEU | 1103 |
| A | SER | 1046 | B | SER | 1140 |
| A | PHE | 1047 | B | VAL | 1101 |
| A | HIS | 1048 | B | SER | 1137 |
| A | ILE | 1049 | B | VAL | 1099 |
| A | SER | 1050 | B | THR | 1135 |
| A | GLN | 1083 | B | GLN | 1120 |
| A | GLY | 1084 | B | ARG | 1108 |
| A | GLY | 1085 | B | ARG | 1108 |
| A | LEU | 1087 | B | ALA | 1106 |
| A | SER | 1090 | B | THR | 1104 |
| A | LYS | 1093 | B | GLN | 1102 |
| A | ARG | 1095 | B | VAL | 1100 |
| A | GLY | 1097 | B | SER | 1098 |
