# Supplementary material for: Evaluation of peptide designing strategy against subunit reassociation in mucin 1: A steered molecular dynamics approach
Source: PLoS One. 2017 Aug 17;12(8):e0183041. doi: 10.1371/journal.pone.0183041 (PMC5560680; doi:10.1371/journal.pone.0183041)
Supplement: S4 Table — (DOCX) [file pone.0183041.s009.docx]

**S4 Table. Top scoring peptide solutions of PEP41 mutants**

| **Peptide** | **Global energy** | **Attractive VdW^a^** | **Repulsive VdW^a^** | **ACE^b^** | **HB^c^** |
| --- | --- | --- | --- | --- | --- |
| PEP41 | -116.75 | -47.64 | 4.8 | -27.77 | -8.27 |
| Mutants | | | | | |
| 2I | -161.84 | -52.26 | 7.32 | -39.39 | -8.11 |
| 4K | -130.18 | -48 | 1.09 | -27.76 | -7.45 |
| 2P | -149.35 | -50.68 | 1.69 | -32.49 | -6.98 |
| 1S | -140.2 | -59.33 | 47.81 | -41.92 | -6.45 |
| 3K | -135.08 | -47.33 | 0.63 | -34.92 | -5.2 |
| Mutant combinations | | | | | |
| 2I4K | -171.47 | -56.38 | 1.39 | -38.64 | -7.26 |
| 2P4K | -88.52 | -48.12 | 71.97 | -33.21 | -8.19 |

^a^Vanderwaals, ^b^Atomic Contact Energy, ^c^Hydrogen Bond. Peptide names represent the position of mutation followed by the single letter code of the substituted aminoacid
