# Supplementary material for: Evaluation of peptide designing strategy against subunit reassociation in mucin 1: A steered molecular dynamics approach
Source: PLoS One. 2017 Aug 17;12(8):e0183041. doi: 10.1371/journal.pone.0183041 (PMC5560680; doi:10.1371/journal.pone.0183041)
Supplement: S5 Table — (DOCX) [file pone.0183041.s010.docx]

**S5 Table. Peptide residues disassociation patterns**

| **Residues** | | **Frames between start and end of peptide disassociation** | | | | | | | | | | | | | | | | | | | |
| --- | --- | --- | --- | --- | --- | --- | --- | --- | --- | --- | --- | --- | --- | --- | --- | --- | --- | --- | --- | --- | --- |
|  | **REF** | **1** | **2** | **3** | **5** | **10** | **20** | **30** | **40** | **50** | **51** | **60** |  |  |  |  |  |  |  |  |  |
| 1 | SER | + | + | + | + | + | + | + | + | + | + | - |  |  |  |  |  |  |  |  |  |
| 2 | VAL | + | + | + | + | + | + | + | - | + | - | - |  |  |  |  |  |  |  |  |  |
| 3 | VAL | + | + | + | + | + | + | + | + | - | - | - |  |  |  |  |  |  |  |  |  |
| 4 | VAL | + | + | - | + | + | + | + | + | + | - | - |  |  |  |  |  |  |  |  |  |
| 5 | GLN | + | + | - | - | - | - | - | - | - | - | - |  |  |  |  |  |  |  |  |  |
| 6 | LEU | + | - | - | - | - | - | - | - | - | - | - |  |  |  |  |  |  |  |  |  |
| 7 | THR | + | + | - | - | - | - | - | - | - | - | - |  |  |  |  |  |  |  |  |  |
| 8 | LEU | - | - | - | - | - | - | - | - | - | - | - |  |  |  |  |  |  |  |  |  |
| 9 | ALA | - | - | - | - | - | - | - | - | - | - | - |  |  |  |  |  |  |  |  |  |
|  |  |  |  |  |  |  |  |  |  |  |  |  |  |  |  |  |  |  |  |  |  |
|  | **BE1** | **1** | **3** | **5** | **15** | **20** | **30** | **40** | **50** | **60** | **75** | **86** |  |  |  |  |  |  |  |  |  |
| 1 | GLY | + | + | + | + | + | + | - | + | - | + | + |  |  |  |  |  |  |  |  |  |
| 2 | HIS | + | + | + | + | - | + | + | + | + | - | - |  |  |  |  |  |  |  |  |  |
| 3 | GLN | + | + | + | - | - | + | + | + | + | - | - |  |  |  |  |  |  |  |  |  |
| 4 | TRP | + | + | - | - | - | - | - | - | - | - | - |  |  |  |  |  |  |  |  |  |
| 5 | PHE | - | - | - | - | - | - | - | - | - | - | - |  |  |  |  |  |  |  |  |  |
| 6 | ARG | + | + | - | - | - | - | - | - | - | - | - |  |  |  |  |  |  |  |  |  |
| 7 | PHE | + | + | - | - | - | - | - | - | - | - | - |  |  |  |  |  |  |  |  |  |
| 8 | GLY | + | - | - | - | - | - | - | - | - | - | - |  |  |  |  |  |  |  |  |  |
| 9 | PHE | - | - | - | - | - | - | - | - | - | - | - |  |  |  |  |  |  |  |  |  |
|  |  |  |  |  |  |  |  |  |  |  |  |  |  |  |  |  |  |  |  |  |  |
|  | **BE2** | **1** | **2** | **3** | **4** | **10** | **15** | **40** | **80** | **105** | **180** | **200** | **225** | **230** | **265** | **270** | **275** | **278** | **300** | **302** | **310** |
| 1 | PRO | + | + | + | + | + | + | + | + | + | + | + | - | - | - | + | + | - | + | + | - |
| 2 | HIS | + | + | + | + | + | + | + | + | + | + | + | + | + | + | + | + | + | - | - | - |
| 3 | CYS | + | + | - | + | + | + | + | - | + | + | + | + | - | + | + | + | - | - | - | - |
| 4 | TRP | - | - | - | + | + | + | + | + | - | - | - | - | + | - | - | - | - | - | - | - |
| 5 | TRP | - | - | - | - | - | - | - | - | - | + | - | + | - | - | - | - | - | - | - | - |
| 6 | HIS | - | - | + | - | - | + | + | + | + | - | - | - | - | - | - | - | - | - | - | - |
| 7 | TRP | - | - | - | - | - | - | - | - | - | - | - | - | - | - | - | - | - | - | - | - |
| 8 | VAL | - | - | - | - | - | - | - | - | - | - | - | - | - | - | - | - | - | - | - | - |
| 9 | PHE | + | - | - | - | - | - | - | - | - | - | - | - | - | - | - | - | - | - | - | - |
|  |  |  |  |  |  |  |  |  |  |  |  |  |  |  |  |  |  |  |  |  |  |
|  | **BE3** | **1** | **5** | **10** | **15** | **16** | **17** | **19** | **20** | **35** | **55** | **57** |  |  |  |  |  |  |  |  |  |
| 1 | PRO | - | - | - | - | - | - | - | - | - | - | - |  |  |  |  |  |  |  |  |  |
| 2 | HIS | + | + | + | + | + | + | - | - | - | - | - |  |  |  |  |  |  |  |  |  |
| 3 | CYS | - | - | - | - | - | - | - | - | + | + | - |  |  |  |  |  |  |  |  |  |
| 4 | TRP | - | - | - | + | - | + | - | + | + | + | - |  |  |  |  |  |  |  |  |  |
| 5 | TRP | - | - | - | - | - | - | - | - | - | - | - |  |  |  |  |  |  |  |  |  |
| 6 | LEU | - | - | - | - | - | - | - | - | - | - | - |  |  |  |  |  |  |  |  |  |
| 7 | TRP | + | + | - | - | - | - | - | - | - | - | - |  |  |  |  |  |  |  |  |  |
| 8 | VAL | - | - | - | - | - | - | - | - | - | - | - |  |  |  |  |  |  |  |  |  |
| 9 | PHE | + | + | + | - | + | + | + | - | - | - | - |  |  |  |  |  |  |  |  |  |
|  |  |  |  |  |  |  |  |  |  |  |  |  |  |  |  |  |  |  |  |  |  |
|  | **HB1** | **1** | **5** | **10** | **20** | **30** | **35** | **40** | **60** | **65** | **70** | **83** |  |  |  |  |  |  |  |  |  |
| 1 | GLN | + | + | - | - | - | - | - | - | - | - | - |  |  |  |  |  |  |  |  |  |
| 2 | GLY | - | - | - | - | - | - | - | - | - | - | - |  |  |  |  |  |  |  |  |  |
| 3 | HIS | + | + | + | + | - | + | - | - | - | - | - |  |  |  |  |  |  |  |  |  |
| 4 | ARG | + | + | + | + | - | - | - | - | - | - | - |  |  |  |  |  |  |  |  |  |
| 5 | PHE | - | - | - | - | - | - | - | - | - | - | - |  |  |  |  |  |  |  |  |  |
| 6 | TRP | + | + | + | + | + | - | - | - | - | - | - |  |  |  |  |  |  |  |  |  |
| 7 | PHE | - | - | - | - | - | - | - | - | - | - | - |  |  |  |  |  |  |  |  |  |
| 8 | PHE | + | + | + | + | + | + | + | + | - | - | - |  |  |  |  |  |  |  |  |  |
| 9 | GLY | + | + | + | + | + | + | + | + | + | + | - |  |  |  |  |  |  |  |  |  |
|  |  |  |  |  |  |  |  |  |  |  |  |  |  |  |  |  |  |  |  |  |  |
|  | **HB2** | **1** | **3** | **5** | **10** | **15** | **20** | **30** | **35** | **40** |  |  |  |  |  |  |  |  |  |  |  |
| 1 | GLN | + | + | + | + | + | + | - | - | - |  |  |  |  |  |  |  |  |  |  |  |
| 2 | PRO | - | - | - | - | - | - | - | - | - |  |  |  |  |  |  |  |  |  |  |  |
| 3 | HIS | + | - | - | - | + | - | + | + | - |  |  |  |  |  |  |  |  |  |  |  |
| 4 | LYS | - | - | - | - | - | - | - | - | - |  |  |  |  |  |  |  |  |  |  |  |
| 5 | PHE | - | - | + | + | + | - | - | - | - |  |  |  |  |  |  |  |  |  |  |  |
| 6 | TRP | + | + | - | + | + | - | - | - | - |  |  |  |  |  |  |  |  |  |  |  |
| 7 | PHE | + | + | - | - | - | - | - | - | - |  |  |  |  |  |  |  |  |  |  |  |
| 8 | PHE | + | + | - | - | - | - | - | - | - |  |  |  |  |  |  |  |  |  |  |  |
| 9 | GLY | + | + | - | - | - | - | - | - | - |  |  |  |  |  |  |  |  |  |  |  |
|  |  |  |  |  |  |  |  |  |  |  |  |  |  |  |  |  |  |  |  |  |  |
|  | **PRP** | **1** | **5** | **10** | **15** | **25** | **30** | **35** | **40** | **167** |  |  |  |  |  |  |  |  |  |  |  |
| 1 | LYS | - | - | - | - | - | - | - | - | - |  |  |  |  |  |  |  |  |  |  |  |
| 2 | ASN | - | + | - | - | - | - | - | - | - |  |  |  |  |  |  |  |  |  |  |  |
| 3 | CYS | + | + | + | + | + | + | - | - | - |  |  |  |  |  |  |  |  |  |  |  |
| 4 | TYR | - | - | - | - | - | - | + | - | - |  |  |  |  |  |  |  |  |  |  |  |
| 5 | LEU | + | + | + | + | + | + | - | - | - |  |  |  |  |  |  |  |  |  |  |  |
| 6 | TRP | - | - | - | - | - | - | - | - | - |  |  |  |  |  |  |  |  |  |  |  |
| 7 | PHE | + | - | + | + | - | - | - | - | - |  |  |  |  |  |  |  |  |  |  |  |
| 8 | ILE | + | - | - | - | + | - | - | - | - |  |  |  |  |  |  |  |  |  |  |  |
| 9 | VAL | + | - | + | + | - | - | - | - | - |  |  |  |  |  |  |  |  |  |  |  |
| 10 | ARG | + | + | + | + | + | + | - | + | + |  |  |  |  |  |  |  |  |  |  |  |
| 11 | THR | + | + | + | + | + | + | + | + | + |  |  |  |  |  |  |  |  |  |  |  |
|  |  |  |  |  |  |  |  |  |  |  |  |  |  |  |  |  |  |  |  |  |  |
|  | **RB1** | **1** | **3** | **5** | **10** | **40** | **45** | **56** | **73** |  |  |  |  |  |  |  |  |  |  |  |  |
| 1 | SER | + | + | + | + | + | + | + | - |  |  |  |  |  |  |  |  |  |  |  |  |
| 2 | CYS | - | - | - | - | - | - | - | - |  |  |  |  |  |  |  |  |  |  |  |  |
| 3 | PRO | - | - | - | - | - | - | - | - |  |  |  |  |  |  |  |  |  |  |  |  |
| 4 | LEU | + | + | + | + | + | + | - | - |  |  |  |  |  |  |  |  |  |  |  |  |
| 5 | SER | + | + | + | + | + | - | - | - |  |  |  |  |  |  |  |  |  |  |  |  |
| 6 | LEU | + | + | - | - | - | - | - | - |  |  |  |  |  |  |  |  |  |  |  |  |
| 7 | CYS | + | - | - | - | - | - | - | - |  |  |  |  |  |  |  |  |  |  |  |  |
| 8 | LEU | + | - | - | - | - | - | - | - |  |  |  |  |  |  |  |  |  |  |  |  |
| 9 | VAL | + | - | - | - | - | - | - | - |  |  |  |  |  |  |  |  |  |  |  |  |
|  |  |  |  |  |  |  |  |  |  |  |  |  |  |  |  |  |  |  |  |  |  |
|  | **RB2** | **1** | **33** | **35** | **30** | **40** | **45** | **100** | **120** | **123** | **125** | **127** |  |  |  |  |  |  |  |  |  |
| 1 | SER | + | + | + | + | + | - | - | - | - | - | - |  |  |  |  |  |  |  |  |  |
| 2 | CYS | + | - | - | - | - | - | - | - | - | - | - |  |  |  |  |  |  |  |  |  |
| 3 | GLY | + | + | + | - | - | - | - | - | - | - | - |  |  |  |  |  |  |  |  |  |
| 4 | LEU | + | + | + | + | + | + | + | - | - | - | - |  |  |  |  |  |  |  |  |  |
| 5 | SER | + | + | + | + | + | + | + | - | - | - | - |  |  |  |  |  |  |  |  |  |
| 6 | LEU | + | + | + | + | + | + | + | + | - | - | - |  |  |  |  |  |  |  |  |  |
| 7 | CYS | - | - | - | - | - | - | - | + | + | + | - |  |  |  |  |  |  |  |  |  |
| 8 | LEU | + | + | - | + | + | + | + | + | + | - | - |  |  |  |  |  |  |  |  |  |
| 9 | TRP | + | + | + | + | + | + | + | + | - | + | + |  |  |  |  |  |  |  |  |  |
|  |  |  |  |  |  |  |  |  |  |  |  |  |  |  |  |  |  |  |  |  |  |
|  | **RB3** | **1** | **2** | **4** | **10** | **20** | **35** | **40** | **43** | **44** | **46** |  |  |  |  |  |  |  |  |  |  |
| 1 | CYS | + | + | - | - | - | - | - | - | - | - |  |  |  |  |  |  |  |  |  |  |
| 2 | CYS | + | - | - | - | - | - | - | - | - | - |  |  |  |  |  |  |  |  |  |  |
| 3 | VAL | + | - | - | - | - | - | - | - | - | - |  |  |  |  |  |  |  |  |  |  |
| 4 | LEU | + | + | + | + | - | - | - | - | - | - |  |  |  |  |  |  |  |  |  |  |
| 5 | SER | + | + | + | + | - | - | - | - | - | - |  |  |  |  |  |  |  |  |  |  |
| 6 | LEU | + | + | + | + | + | + | + | - | - | - |  |  |  |  |  |  |  |  |  |  |
| 7 | CYS | + | + | + | + | + | - | - | + | - | - |  |  |  |  |  |  |  |  |  |  |
| 8 | LEU | + | + | + | + | + | + | + | + | - | - |  |  |  |  |  |  |  |  |  |  |
| 9 | VAL | + | + | + | + | + | + | + | + | + | - |  |  |  |  |  |  |  |  |  |  |

“+” indicates the residue is bonded to the receptor, “-” residue disassociated from receptor
