# Supplementary material for: Evaluation of peptide designing strategy against subunit reassociation in mucin 1: A steered molecular dynamics approach
Source: PLoS One. 2017 Aug 17;12(8):e0183041. doi: 10.1371/journal.pone.0183041 (PMC5560680; doi:10.1371/journal.pone.0183041)
Supplement: S6 Table — (DOCX) [file pone.0183041.s011.docx]

**S6 Table. Peptide residue contribution towards protein-peptide interaction**

| **Peptide_residues** | **MM^a^** | **Polar^b^** | **APolar^c^** | **Total** |
| --- | --- | --- | --- | --- |
| **BE1** | | | | |
| GLY-1 | -135.4627 | 117.368 | -2.5831 | -20.7107 |
| HIS-2 | -50.1405 | 25.7823 | -4.1937 | -28.5686 |
| GLN-3 | -30.0286 | 19.2525 | -2.836 | -13.594 |
| TRP-4 | -62.2545 | 27.3078 | -5.1228 | -40.0787 |
| PHE-5 | -28.4751 | 14.3249 | -2.842 | -16.9934 |
| ARG-6 | -37.8077 | 20.9159 | -2.8383 | -19.7132 |
| PHE-7 | -10.9816 | 3.3643 | -1.6154 | -9.2199 |
| GLY-8 | -7.415 | 4.9606 | -0.9432 | -3.3874 |
| PHE-9 | -69.6775 | 116.0625 | -1.4068 | 44.9219 |
| **BE2** | | | | |
| PRO-1 | -154.3574 | 111.3564 | -4.5269 | -47.5331 |
| HIS-2 | -46.3705 | 23.1559 | -3.3205 | -26.5322 |
| CYS-3 | -28.707 | 12.6945 | -2.5614 | -18.5803 |
| TRP-4 | -36.9348 | 16.7085 | -3.3927 | -23.6171 |
| TRP-5 | -47.3405 | 15.6214 | -4.2868 | -36.023 |
| HIS-6 | -30.6429 | 20.9237 | -3.0451 | -12.7473 |
| TRP-7 | -29.6533 | 16.2241 | -3.0572 | -16.4781 |
| VAL-8 | -11.8906 | 4.6568 | -1.6234 | -8.8526 |
| PHE-9 | -18.8123 | 22.9874 | -2.166 | 2 |
| **BE3** | | | | |
| PRO-1 | -83.3936 | 52.7638 | -1.9055 | -32.4681 |
| HIS-2 | -28.4833 | 18.4567 | -2.5536 | -12.5751 |
| CYS-3 | -20.2406 | 11.6348 | -1.0154 | -9.6268 |
| TRP-4 | -46.9336 | 24.5485 | -5.9059 | -28.2809 |
| TRP-5 | -38.6951 | 17.4141 | -4.8741 | -26.1532 |
| LEU-6 | -23.3565 | 7.831 | -2.885 | -18.4096 |
| TRP-7 | -35.148 | 13.7639 | -3.7093 | -25.0872 |
| VAL-8 | -15.4963 | 7.0731 | -1.588 | -9.9949 |
| PHE-9 | -54.5251 | 90.9214 | -4.4042 | 32.0042 |
| **HB1** | | | | |
| GLN-1 | -49.0336 | 31.1176 | -2.6822 | -20.5962 |
| GLY-2 | -5.1481 | 2.2374 | -0.6956 | -3.6151 |
| HIS-3 | -26.6639 | 14.5222 | -2.3747 | -14.5145 |
| ARG-4 | -24.4838 | 10.4926 | -1.5521 | -15.5726 |
| PHE-5 | -21.511 | 7.7441 | -2.0612 | -15.8256 |
| TRP-6 | -42.5702 | 14.1044 | -3.979 | -32.439 |
| PHE-7 | -39.7875 | 15.0646 | -4.0316 | -28.7433 |
| PHE-8 | -55.2358 | 22.9939 | -5.2612 | -37.4988 |
| GLY-9 | 37.4713 | 22.7002 | -2.1903 | 58.0248 |
| **HB2** | | | | |
| GLN-1 | -53.1212 | 48.2239 | -2.5453 | -7.4427 |
| PRO-2 | -13.5935 | -0.6886 | -1.9907 | -16.2743 |
| HIS-3 | -32.725 | 20.2777 | -2.8761 | -15.2844 |
| LYS-4 | -33.1626 | 15.373 | -2.0643 | -19.9596 |
| PHE-5 | -21.9354 | 7.2412 | -1.9986 | -16.6904 |
| TRP-6 | -45.0502 | 17.5568 | -3.3475 | -30.743 |
| PHE-7 | -28.6789 | 13.3379 | -2.7706 | -18.1725 |
| PHE-8 | -43.4858 | 19.3072 | -3.0666 | -27.2145 |
| GLY-9 | 17.4169 | 36.2659 | -2.0681 | 51.6321 |
| **RB1** | | | | |
| SER-1 | -151.8374 | 105.7657 | -2.7506 | -48.8815 |
| CYS-2 | -22.1253 | 11.3971 | -2.1437 | -12.8722 |
| PRO-3 | -36.8913 | 9.9977 | -3.6599 | -30.5461 |
| LEU-4 | -40.1845 | 9.9551 | -3.0715 | -33.3028 |
| SER-5 | -28.4343 | 20.2101 | -2.4906 | -10.7256 |
| LEU-6 | -40.9682 | 11.3962 | -2.899 | -32.4699 |
| CYS-7 | -32.9546 | 11.9787 | -2.299 | -23.2846 |
| LEU-8 | -42.2603 | 8.8537 | -3.9562 | -37.3586 |
| VAL-9 | -84.6968 | 125.6207 | -3.6205 | 37.3688 |
| **RB2** | | | | |
| SER-1 | -140.8597 | 125.2473 | -3.4997 | -19.0748 |
| CYS-2 | -35.9758 | 15.5374 | -2.6995 | -23.1243 |
| GLY-3 | -18.0967 | 11.3278 | -1.6229 | -8.3751 |
| LEU-4 | -43.4777 | 12.9577 | -3.5973 | -34.1171 |
| SER-5 | -34.235 | 23.1404 | -2.6643 | -13.7793 |
| LEU-6 | -44.9253 | 12.527 | -3.2582 | -35.6466 |
| CYS-7 | -33.8679 | 13.1603 | -2.7958 | -23.5217 |
| LEU-8 | -36.4663 | 15.927 | -2.749 | -23.301 |
| TRP-9 | -61.2835 | 67.6431 | -2.335 | 4.0681 |
| **RB3** | | | | |
| CYS-1 | -185.8677 | 152.0356 | -4.2338 | -38.0386 |
| CYS-2 | -30.8191 | 16.4106 | -2.3722 | -16.7881 |
| VAL-3 | -27.755 | 9.0093 | -2.5624 | -21.3103 |
| LEU-4 | -44.774 | 11.7303 | -3.6213 | -36.657 |
| SER-5 | -24.8372 | 17.2547 | -1.8954 | -9.4756 |
| LEU-6 | -43.5137 | 12.9227 | -3.0099 | -33.6138 |
| CYS-7 | -35.2667 | 14.0256 | -2.4123 | -23.6584 |
| LEU-8 | -41.6961 | 12.0968 | -3.8368 | -33.4284 |
| VAL-9 | -44.4357 | 71.292 | -3.1814 | 23.6231 |
| **PRP** | | | | |
| LYS-1 | -16.4178 | 13.9758 | -1.3181 | -3.7285 |
| ASN-2 | -17.1583 | 14.0118 | -1.9969 | -5.1327 |
| CYS-3 | -22.1147 | 14.4696 | -1.935 | -9.5685 |
| TYR-4 | -32.9127 | 14.7125 | -2.9644 | -21.1861 |
| LEU-5 | -25.1398 | 8.0355 | -2.3629 | -19.4681 |
| TRP-6 | -43.6066 | 20.3749 | -4.1458 | -27.4267 |
| PHE-7 | -29.2131 | 11.5484 | -2.8496 | -20.531 |
| ILE-8 | -39.0813 | 13.4044 | -3.3141 | -29.0116 |
| VAL-9 | -33.3614 | 12.0855 | -2.6309 | -23.8968 |
| ARG-10 | -160.4135 | 107.5379 | -5.4439 | -58.2599 |
| THR-11 | 15.962 | 64.4636 | -3.5544 | 76.8942 |
| **REF** | | | | |
| SER-1098 | -156.0176 | 123.0889 | -3.5102 | -36.3691 |
| VAL-1099 | -41.7171 | 7.3834 | -3.3841 | -37.6956 |
| VAL-1100 | -31.7849 | 8.7467 | -2.2888 | -25.3246 |
| VAL-1101 | -38.4119 | 9.9394 | -2.7312 | -31.2042 |
| GLN-1102 | -35.9021 | 25.5701 | -2.8701 | -13.2259 |
| LEU-1103 | -42.1267 | 11.7509 | -3.1183 | -33.5084 |
| THR-1104 | -31.9842 | 18.0849 | -3.0989 | -16.9732 |
| LEU-1105 | -38.5728 | 12.0719 | -3.9803 | -30.4823 |
| ALA-1106 | -63.3689 | 97.2656 | -2.7213 | 31.3019 |
| **PF** | | | | |
| PRO-1 | -42.0866 | 42.9927 | -3.7917 | -2.9006 |
| HIS-2 | -45.6299 | 19.5735 | -3.4314 | -29.4892 |
| CYS-3 | -35.6775 | 14.2308 | -2.2513 | -23.7054 |
| LEU-4 | -32.9964 | 8.8533 | -2.7953 | -26.9495 |
| SER-5 | -25.8901 | 17.6458 | -1.4808 | -9.7214 |
| TRP-6 | -35.0036 | 17.7611 | -3.4927 | -20.7503 |
| CYS-7 | -22.864 | 14.1413 | -2.0055 | -10.7289 |
| PHE-8 | -37.3132 | 16.3296 | -3.4621 | -24.4462 |
| TRP-9 | 17.8366 | 19.9616 | -5.8261 | 31.966 |

^a^Molecular mechanics energy; ^b^Polar solvation energy; ^c^Non-polar solvation energy
